# Prescribing Prevalence, Effectiveness, and Mental Health Safety of Smoking Cessation Medicines in Patients With Mental Disorders

**DOI:** 10.1093/ntr/ntz072

**Published:** 2019-07-10

**Authors:** Gemma M J Taylor, Taha Itani, Kyla H Thomas, Dheeraj Rai, Tim Jones, Frank Windmeijer, Richard M Martin, Marcus R Munafò, Neil M Davies, Amy E Taylor

**Affiliations:** 1 Medical Research Council Integrative Epidemiology Unit, Bristol Medical School, Population Health Sciences, University of Bristol, Bristol, UK; 2 UK Centre for Tobacco and Alcohol Studies, School of Psychological Science, University of Bristol, Bristol, UK; 3 Addiction and Mental Health Group (AIM), Department of Psychology, University of Bath, Bath, UK; 4 Bristol Medical School, Population Health Sciences, Canynge Hall, University of Bristol, Bristol, UK; 5 Centre for Academic Mental Health; Bristol Medical School, Population Health Sciences, University of Bristol, Bristol, UK; 6 National Institute for Health Research Collaboration for Leadership in Applied Health Research and Care West (NIHR CLAHRC West) at University Hospitals Bristol NHS Foundation Trust, Bristol, UK; 7 Department of Economics, University of Bristol, Bristol, UK; 8 NIHR Biomedical Research Centre at the University Hospitals Bristol NHS Foundation Trust and the University of Bristol, Bristol, UK

## Abstract

**Objective:**

We conducted a prospective cohort study of the Clinical Practice Research Database to estimate rates of varenicline and nicotine replacement therapy (NRT) prescribing and the relative effects on smoking cessation, and mental health.

**Methods:**

We used multivariable logistic regression, propensity score matched regression, and instrumental variable analysis. Exposure was varenicline or NRT prescription. Mental disorders were bipolar, depression, neurotic disorder, schizophrenia, or prescriptions of antidepressants, antipsychotics, hypnotics/anxiolytics, mood stabilizers. Outcomes were smoking cessation, and incidence of neurotic disorder, depression, prescription of antidepressants, or hypnotics/anxiolytics. Follow-ups were 3, 6, and 9 months, and at 1, 2, and 4 years.

**Results:**

In all patients, NRT and varenicline prescribing declined during the study period. Seventy-eight thousand four hundred fifty-seven smokers with mental disorders aged ≥18 years were prescribed NRT (*N* = 59 340) or varenicline (*N* = 19 117) from September 1, 2006 to December 31, 2015. Compared with smokers without mental disorders, smokers with mental disorders had 31% (95% CI: 29% to 33%) lower odds of being prescribed varenicline relative to NRT, but had 19% (95% CI: 15% to 24%) greater odds of quitting at 2 years when prescribed varenicline relative to NRT. Overall, varenicline was associated with decreased or similar odds of worse mental health outcomes than NRT in patients both with and without mental disorders, although there was some variation when analyses were stratified by mental disorder subgroup.

**Conclusions:**

Smoking cessation medication prescribing may be declining in primary care. Varenicline was more effective than NRT for smoking cessation in patients with mental disorders and there is not clear consistent evidence that varenicline is adversely associated with poorer mental health outcomes.

**Implications:**

Patients with mental disorders were less likely to be prescribed varenicline than NRT. We triangulated results from three analytical techniques. We found that varenicline was more effective than NRT for smoking cessation in patients with mental disorders. Varenicline was generally associated with similar or decreased odds of poorer mental health outcomes (ie, improvements in mental health) when compared with NRT. We report these findings cautiously as our data are observational and are at risk of confounding.

## Background

Smoking is the world’s leading cause of preventable illness and death.^1^ One in every two smokers will die because of their addiction unless they quit.^2^ In many countries, such as the United Kingdom, smoking prevalence has decreased from 46% during 1970s to about 16% in recent years.^3^ However, smoking prevalence has not changed to the same extent in people with mental disorders. Smoking prevalence in people with mental disorders was estimated at around twice the rate of the general population at 40% in 2010/2011.^4^ People with mental disorders smoke more cigarettes per day, are more heavily addicted, and are more likely to relapse than are the general population.^5^ The burden of smoking-related mortality and morbidity in people with mental disorders is clear, with estimates of lowered life expectancy of up to 18 years when compared with the general population.^6^

Data from Cochrane Group meta-analyses of randomized controlled trials (RCTs),^7^ and from recent observational studies have found that varenicline produces higher quit rates compared with nicotine replacement therapy (NRT) in the general population.^8^ However, there has been controversy around the neuropsychiatric safety of this drug and therefore clinicians may be reluctant to prescribe varenicline to smokers with mental disorders. However, observational studies and RCTs show little evidence that varenicline increases risks of neuropsychiatric harms in the general population.^7,9^

There are few studies examining the effectiveness of varenicline and varenicline’s association with mental health outcomes in people with mental disorders. Molero et al.^10^ reported an association between varenicline and increased risk of mood and anxiety conditions in people with psychiatric conditions, hazard ratio for anxiety conditions, 1.23 (95% CI: 1.01% to 1.51%) and mood conditions, 1.31 (95% CI: 1.06% to 1.63%), however they did not stratify analyses by mental disorder (eg, schizophrenia). The EAGLES RCT compared NRT and varenicline for smoking cessation and neuropsychiatric safety in a psychiatric cohort,^11^ and reported that varenicline produced higher abstinence rates compared with NRT at 9- to 24-weeks follow-up, odds ratio 1.51 (95% CI: 1.19% to 1.93%), and found little evidence that varenicline caused neuropsychiatric harm when compared to NRT, risk difference 1.22 (95% CI: −0.81% to 3.25%).^11^ However, the trial’s longest follow-up was 24 weeks and was underpowered to detect rare events, particularly when stratified by mental diagnosis.

Therefore, in this study in the Clinical Practice Research Database (CPRD) we aimed to:

1. Describe rates of smoking cessation medication prescribing, and smoking prevalence stratified by mental disorder in primary care from 2006 to 2015.
2. Estimate the relative effectiveness of varenicline and NRT on smoking cessation in patients with mental disorders, compared with those without mental disorders, at 3, 6, 9 months and 1, 2, 4 years after first prescription.
3. Determine the association between varenicline or NRT and subsequent diagnosis of depression, neurotic disorder, prescription of anti-depressants, or hypnotics/anxiolytics in patients with mental disorders, compared with those without mental disorders, at 3, 6, 9 months and 1, 2, 4 years after first prescription.

Because we were concerned about confounding we triangulated results across three different analytical methods^12^: multivariable logistic regression, propensity score matched regression, and instrumental variable analysis.

## Methods

We conducted a prospective cohort study using electronic medical records from 654 general practices in England from 2006 onwards.^13^ It was approved by the Independent Scientific Advisory Committee for MHRA Database Research (www.cprd.com/isac/).

### Data Source and Population

We obtained data from the Clinical Practice Research Database (CPRD) (www.cprd.com) which contains data on more than 13 million UK primary care patients. Registered patients are representative of the United Kingdom’s population.^14^ CPRD data have been validated, audited, and quality checked.^15^

### Code Lists

We defined variables using medical and product codes within the CPRD. Code lists were developing using a list from a previously published study^16^ and then agreed upon by field experts (DR and KHT), and by using the British National Formulary (BNF) and the International Classification of Diseases (ICD-10).

### Patients

We included smokers and nonsmokers for smoking prevalence estimates, and we included smokers prescribed either varenicline or NRT for effectiveness/safety estimates. For all aims, we included patients aged ≥18 years, with no breaks in their electronic medical records, who had complete information on year of birth, registration date, and sex. For the effectiveness/safety analysis, we excluded patients who registered with their general practice within 365 days of their first recorded varenicline/NRT prescription to ensure availability of baseline and exposure data.

Patients were categorized as having a mental disorder if: (1) they had been diagnosed with one of the following common mental disorders 365 days before first varenicline/NRT prescription: depression (F32–F39), neurotic disorder (F40–F48), (2) if they had a lifetime record of bipolar disorder (F30–F31), or schizophrenia or other nonaffective psychotic disorders (F20–F29), or (3) if they were prescribed any of the following medications 365 days before smoking cessation medication prescription: antidepressants (BNF chapter 4.3), antipsychotics (4.2.1.–4.2.2), hypnotics or anxiolytics (4.1), or mood stabilizers (4.2.3). Patients with no record of the above listed mental disorders or psychoactive medication prescriptions were considered to have no mental disorder.

### Variables

#### Defining Variables for Prevalence of Smoking, and NRT and Varenicline Prescribing Estimates

For smoking prevalence estimates a patient’s smoking status (aged ≥18 years) was defined by a record indicating smoking/nonsmoking or prescription of NRT/varenicline in that year, and restricted to within the registration date for each patient. Each patient’s smoking status was carried forward until there was evidence of a change in smoking status or carried backward if the patient only had smoking status recorded in the final year of their registration period.

Prevalence of prescriptions of varenicline and NRT were calculated by dividing the number of prescriptions each year from 2007 to 2015 (there were very few varenicline prescriptions for patients with mental disorders in 2006) by the number of current smokers in each year. In both instances, prevalence was estimated for people with and without mental disorders.

For both smoking and prescribing prevalence, we generated standardised rates (with 95% CIs) using a direct standardisation method to account for differences in age (ie, 18–24, 25–34, 35–49, 50–59, 60+ years) and sex between groups. We used the CPRD population in 2015 as our standard population for calculating standardised smoking rates, and the CPRD smoker population in 2015 as our standard population for calculating standardised prescribing prevalence rates. Individuals with missing smoking information were excluded from the denominators.

#### Intervention Groups

The intervention was defined as varenicline prescription, and control as NRT prescription (eg, patches, etc.). Prescriptions used to define treatment groups were issued between September 1, 2006 and August 31, 2016, with no prior record of use of a related product in the preceding 18 months. We used the first treatment episode to ensure that intervention groups were “new users” of the medication.^17^ We did not model treatment switching because this is likely to be strongly related to patient characteristics and the resulting pattern of time-dependent confounding-by-indication would be difficult to control for. Intervention was defined using a variable representing NRT (0) or varenicline (1).

#### Outcomes

##### Smoking Cessation.

 This was defined as having an electronic record indicating smoking cessation.^18^ From April 1, 2004 general practitioners (GPs) have been incentivised to opportunistically record patients’ smoking status as current, former or never smoker in their records; these data are repeatedly recorded (ie, at registration and on a regular basis thereafter)^19^ as part of a nationwide incentive program^20^ and these smoking records are consistent with smoking prevalence reported in representative population surveys.^21^ We determined each patient’s smoking status by using their most recent smoking record identified between cohort entry and each follow-up period (eg, 3 and 6 months); that is, the closest smoking record to each follow-up period was selected. For analyses smoking status was defined as smoker (0) or quit (1). Patients with missing smoking data were assumed to be continuing smokers.^22^ This definition has been used previously and is robust to sensitivity tests in which we multiple imputed missing outcome data.^23^

##### Mental Health Safety.

 Safety outcomes were defined as any newly occurring medical record indicating depression, neurotic disorder, or prescription of antidepressants, hypnotics/anxiolytics after prescription of smoking cessation medication. Records were analysed as present (1) or absent (0). In analyses where diagnosis of depression or neurotic disorder were the outcomes, we excluded patients with the diagnoses 365 days before exposure (ie, where depression was the outcome, patients with depression diagnosis 365 days before exposure were excluded). We did this because change in diagnoses are unlikely to be consistently recorded in the CPRD.

#### Covariates

Covariates included patients’ age at time of prescription, sex, year of prescription, days registered in the CPRD, history of mental disorder or psychoactive medication prescriptions, evidence of other psychoactive medication prescription or other less common psychiatric disorders (Supplementary Material 1 for details), drug or alcohol misuse, mean number of GP visits one year prior to first prescription, body mass index (BMI), socioeconomic position (ie, index of multiple deprivation [IMD]) and the Charlson Index (ie, measure of chronic illness).^24^

### Follow-up

Primary follow-up was 2-years after each patient’s first prescription of varenicline or NRT. Patients were also followed-up at 3, 6, and 9 months and 1 and 4 years after first prescription of varenicline or NRT.

### Statistical Analysis

Analyses were conducted using Stata 14 MP. Analytic code is available on GitHub (https://github.com/nmdavies/varenicline-mentalhealth).

#### Smoking and Smoking Cessation Medication Prescribing Prevalence Overtime

Formulae described in Supplementary Material 1 were used to calculate smoking and prescribing prevalence each year from 2007, in patients with and without mental disorders.

#### Varenicline’s Effectiveness for Smoking Cessation Compared with NRT

To investigate the effectiveness of varenicline versus NRT on smoking cessation we conducted: (1) multivariable logistic regressions, (2) propensity score matched logistic regressions, and (3) instrumental variable regressions using physicians’ prescribing preferences as the instrument.^25^

All regression models were estimated using robust standard errors, which allowed for potential clustering of patients within practices. Models were repeated in patients: with no mental disorder, any mental disorder; bipolar, depression, neurotic disorder, or schizophrenia; prescribed any psychoactive medication; prescribed antidepressants, antipsychotics, hypnotics/anxiolytics, or mood stabilizers.

We used Bland–Altman tests to determine if the effectiveness of varenicline differed at 2-years follow-up between patients with or without mental disorders (Supplementary Material 1 for further details).

Multivariable adjusted results can suffer from residual confounding by indication (ie, patients prescribed varenicline might be healthier at baseline than those prescribed NRT).^26^ We addressed this by repeating all analyses using: propensity score matched regression by matching patients on the association between their exposure and baseline characteristics (ie, days of history, sex, age at time of first prescription, BMI, multiple deprivation score, number of GP visits 1 year before first prescription, year of first prescription, comorbidity ever (Charlson Index), alcohol misuse ever, drug misuse ever, bipolar ever, depression ever, neurotic disorder ever, schizophrenia ever, anti-depressants ever, antipsychotics ever, hypnotics ever, mood stabilizers ever, prescription of rare psychotropic medication ever, diagnosis of other mental disorder ever), and instrumental variable regression models using physicians’ prescribing preferences as the instrument.^25^ Instrumental variables are proxies for the exposure and defined by the following characteristics: they are related to the intervention, their association with the outcome has no confounders, and they do not directly affect the outcome except via the intervention.^27^ Physicians’ prescribing preferences for varenicline or NRT have been shown to be suitable instrumental variables.^23^ We used a physician’s prescription to their previous patient as a proxy for their prescribing preference.^25,27^ If the instrumental variable assumptions hold, and multivariable adjusted regression results suffer from residual confounding, then the results will differ. However, instrumental variable methods require large sample sizes, as they have lower power than standard regression models because the instrument only explains some of the variance in prescribed treatment. See Supplementary Material 1 for details of analytical methods.

#### Varenicline’s Mental Health Safety Compared With NRT

To determine the mental health safety of varenicline relative to NRT we repeated the analyses described above, with the following outcomes: diagnosis of depression or neurotic disorder, prescription of antidepressants or hypnotics/anxiolytics (ie, as defined above).

### Missing Data

To increase efficiency and minimize selection bias, we used multiple imputation to impute data in patients missing BMI and IMD values.^28^ Using the ICE command, we produced 20 imputed datasets, and the imputation model included all exposures, covariates, and outcomes.^28^

## Results

### Smoking and Smoking Cessation Medication Prescribing Prevalence Overtime

The age and sex standardised smoking prevalence among people with any mental disorder diagnosis/psychoactive medication prescription consistently decreased from 38.6% and 36.6% in 2006 to 32.0% and 29.9% in 2015 (respectively). These estimates were consistently higher than in people with no mental health diagnosis or prescription (from 14.5% in 2006 to 10.9% in 2015; Supplementary Material 1, eFigure 1). Between 2006 and 2015, smoking prevalence decreased in all mental disorders and similarly in patients who were prescribed any of the psychoactive medications (Supplementary Material 1, eFigure 2).

**Figure 1.**
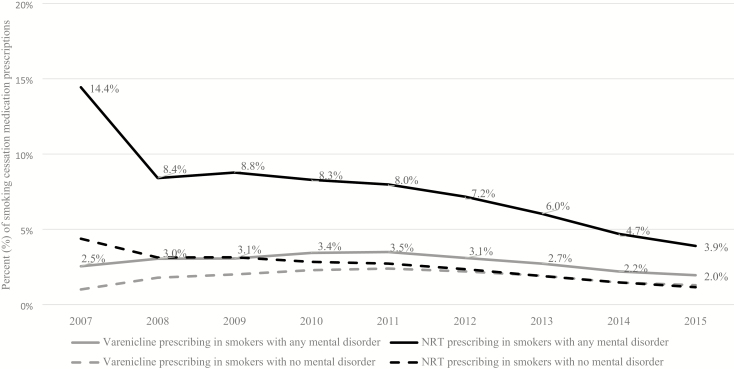
Age and sex standardised percentage (%) of prescriptions of varenicline or NRT in primary care, from 2007 to 2015, in smokers with any mental disorder, compared with smokers with no mental disorder.

Standardised NRT prescribing rates fell between 2007 and 2015 in smokers with and without mental disorders. Varenicline prescribing increased between 2007 and 2011 and then fell between 2011 and 2015 in smokers with and without mental disorders. From 2010 to 2015, both NRT and varenicline prescribing was higher among smokers with mental disorders as compared with those without (Figure 1).

### Varenicline’s Effectiveness and Mental Health Safety Compared With NRT

#### Population Characteristics

There were 361,656 patients prescribed smoking cessation medications between September 1, 2006 and August 31, 2016. Of these 235,314 were eligible for analysis, 159,736 smokers were prescribed NRT and 75,578 prescribed varenicline. Supplementary Material 1, eFigure 3 presents the number of patients excluded with reasons.^18^ Of those prescribed NRT, a range of products were issued: (Supplementary Material 1, eTable 5); 37,009 (23.1%) of the patients prescribed NRT were prescribed more than one type of product. At the time of smoking cessation medicine prescription, the mean age of the participants was 45.8 years (SD = 14.9) and 54% of the cohort were women (Table 1). Baseline data indicated that this cohort was similar to other studies of smokers from the United Kingdom.^4,16,29^

**Table 1.** Baseline Characteristics by Intervention Group, *N* (%)

Characteristic	Whole cohort (*N* = 235 314)	NRT (*N* = 159 736)	Varenicline (*N* = 75 578)
Age at time of first prescription^a^	45.8 (14.9)	46.3 (15.6)	44.4 (13.2)
Sex (female)	116 581 (52.6%)	85 876 (53.8%)	37 883 (50.1%)
Multiple deprivation score (IMD)^b,c^	12	12	12
Number of GP visits 1 year before first prescription^a^	6.9 (7·1)	7·9 (7·4)	6·3 (6·1)
BMI^b,c^	26.5 (0.01)	26.5 (0.02)	26.5 (0.02)
Year of first prescription^d^	2009	2009	2010
Days of history^a^	3089.8 (1934.2)	3054.3 (1908.0)	3164.9 (1986.4)
Comorbidity ever (Charlson Index)	83 888 (35.7%)	59 843 (37.5%)	24 045 (31.8%)
Alcohol misuse ever	18 764 (8.0%)	13 994 (8.8%)	4770 (6.3%)
Drug misuse ever	6431 (2.7%)	4975 (3.1%)	1456 (1.9%)
Self-harm ever	23 960 (10.2%)	17 299 (10.8%)	6661 (8.8%)
Other psychoactive medication ever	640 (<1%)	513 (<1%)	127 (<1%)
Other behavioral/neurologic disorder ever	12 084 (5.1%)	9092 (5.7%)	2992 (4.0%)
Mental disorder diagnosis/prescription			
Any mental disorder diagnosis or psychoactive medication prescription	78 457 (33.3%)	59 340 (37.2%)	19 117 (25.3%)
Bipolar disorder	2012 (<1%)	1799 (1.1%)	213 (<1%)
Depression	17 168 (7.3%)	13 421 (8.4%)	3747 (5.0%)
Neurotic disorder	8394 (3.6%)	6453 (4.0%)	1941 (2.6%)
Schizophrenia and nonaffective psychoses	4704 (2.0%)	4263 (3.7%)	441 (<1%)
Antidepressants	56 756 (24.1%)	43 589 (27.3%)	13 167 (17.4%)
Antipsychotics	11 829 (5.0%)	9843 (6.2%)	1986 (2.6%)
Hypnotics/anxiolytics	31 291 (13.3%)	23 651 (14.8%)	7640 (10.1%)
Mood stabilizers	4728 (2.0%)	4079 (2.6%)	649 (<1%)

^a^Data presented are mean and standard deviation.

^b^Data presented are mean and standard error.

^c^Missing data: BMI data were missing for 14.1% (*N* = 33 059); IMD data were missing for 46.7% (*N* = 109 994), nonimputed data are presented. Missing BMI and IMD values were imputed using multiple imputation.^29^ See Supplement 1 for imputed data (Supplementary Material 1, eTable 2).

^d^Data presented are median.

#### Effectiveness

**Figure 2.**
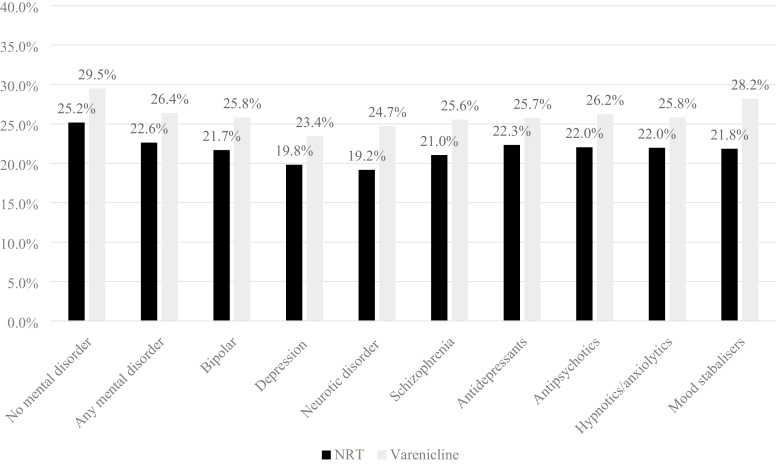
Percentage (%) of patients with an electronic medical record indicating smoking cessation at 2-years follow-up, by exposure and mental disorder.

**Figure 3.**
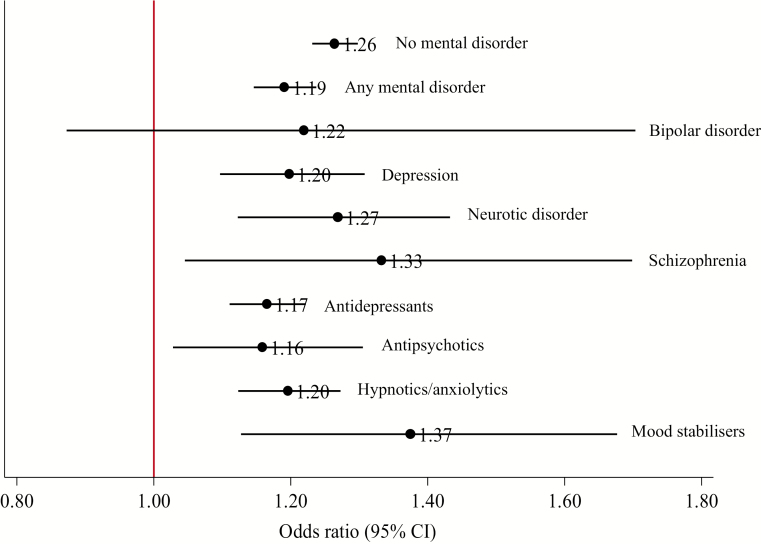
Stratified by mental disorder: The association of prescribing varenicline compared with NRT and smoking cessation at 2-years follow-up. Fully adjusted odds ratios and 95% confidence intervals from logistic regression models. Fully adjusted models were adjusted for: age, sex, days in history, IMD, number of GP visits 1-year before first prescription, BMI, year of first prescription, history of major physical morbidity (Charlson Index), alcohol misuse ever, drug misuse ever, bipolar ever, depression ever, neurotic disorder ever, self-harm ever, other mental disorder ever, antidepressant prescription ever, antipsychotic prescription ever, hypnotics/anxiolytics prescription ever, other psychoactive medication ever, other behavioral/neurologic disorder ever. Missing BMI and IMD values were imputed using multiple imputation.^24^ No mental disorder diagnosis or psychoactive medication prescription (*N* = 156 857); any mental disorder diagnosis or psychoactive medication prescription (*N* = 78 457); bipolar (*N* = 2011); depression (*N* = 17 168); neurotic disorder (*N* = 8394); schizophrenia (*N* = 8,394); antidepressants (*N* = 56 756); antipsychotics (*N* = 11 829); hypnotics/anxiolytics (*N* = 31 291); mood stabilizers (*N* = 4727).

In the cohort, smokers with mental disorders had 31% (95% CI: 29% to 33%) decreased odds of being prescribed varenicline than NRT, compared with smokers without mental disorders (Supplementary Material 1, eTables 3–4). A higher proportion of patients prescribed varenicline had quit smoking at all follow-ups, compared to those prescribed NRT (Figure 2). At 2 years, smokers with mental disorders prescribed varenicline had 19% (95% CI: 15% to 24%) higher odds of quitting smoking (fully adjusted odds ratio) than those prescribed NRT (Figure 3, Supplementary Material 1, eTables 6–8); the association was smaller in patients with mental disorders than in those without mental disorders at 2 years (Bland–Altman *p* value = .02, Supplementary Material 1, eTable 9). The association between varenicline and smoking cessation persisted from 3 months to 4 years but attenuated over time in all patients.

Smokers prescribed varenicline had higher odds of quitting at 2 years for all mental disorder subgroups than those prescribed NRT. However, estimates in patients with bipolar disorder were imprecise (ie, wide confidence intervals). When compared with patients without mental disorders, there was some evidence that the odds of 2-year smoking cessation in those prescribed varenicline compared with NRT was smaller in those prescribed antidepressants (Bland–Altman *p* value = .01, Supplementary Material 1, eTable 9). There was little evidence of heterogeneity for the effectiveness estimates between the other mental disorder groups and patients without mental disorders (all Bland–Altman *p* > .05, Supplementary Material 1, eTable 9). At all other follow-ups, patients prescribed varenicline were more likely to quit compared with NRT (Supplementary Material 1, eTables 7 and 8), although estimates in bipolar and schizophrenia patients were imprecise.

The propensity score matched regression models produced similar results to the multivariable logistic regression models (Supplementary Material 1, eTable 10). The instrumental variable models indicated that smokers with any mental disorder prescribed varenicline were more likely to quit smoking at 2 years, than those prescribed NRT; the risk difference per 100 patients treated was 4.91 (95% CI: 2.42% to 7.40%); this association persisted up to 4 years (Supplementary Material 1, eTables 11 and 12). However, models were imprecise for most subgroups (Supplementary Material 1, eTable 12). There was little evidence of heterogeneity across most mental disorder subgroups (all Bland–Altman *p* > .05, Supplementary Material 1, eTable 13).

#### Mental Health Safety

**Figure 4.**
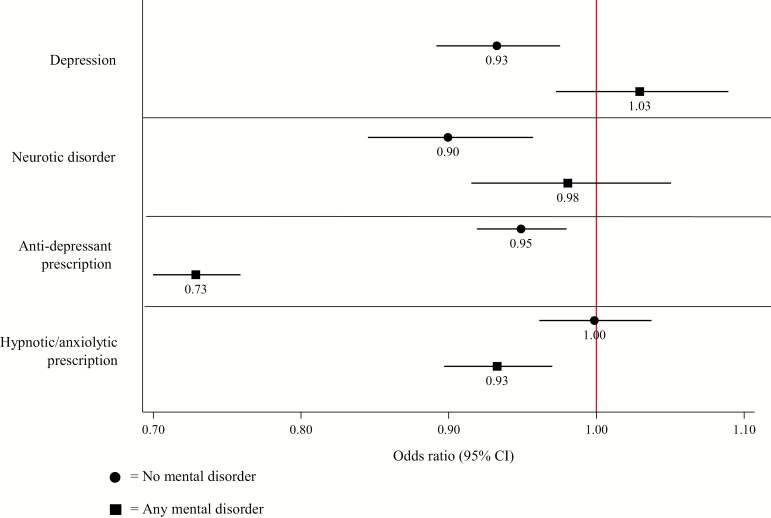
Fully adjusted odds ratio and 95% confidence intervals from logistic regression models for the association between varenicline compared with NRT with depression, neurotic disorder, and prescription of antidepressants or hypnotic/anxiolytics at 2 years, in patients with or without any mental disorder.

At 2 years, patients with any or no mental disorder prescribed varenicline had similar or decreased odds of receiving diagnoses of depression, neurotic disorder, or prescriptions for anti-depressants or hypnotic/anxiolytics than those prescribed NRT (Figure 4). These findings were consistent at all follow-ups and across all three analytical approaches (Supplementary Material 2, eTables 1–24). There was weak evidence that in patients with any mental disorder varenicline was associated with a small increase in depression diagnosis compared with NRT at 4 years and this was consistent across propensity score matched and instrumental variable models. There was evidence that patients with schizophrenia or those prescribed mood stabilizers who were prescribed varenicline were more likely to be diagnosed with depression than those prescribed NRT (Supplementary Material 2, eTables 5, 9 and 17); estimates from propensity score matching and instrumental variable analysis were in the same direction in these groups but were imprecise.

## Discussion

### Main Findings

Smoking prevalence was higher in patients with mental disorders, than in those without, and prescriptions of smoking cessation medications fell during the study period. Although varenicline was more effective than NRT for long-term smoking cessation in patients with mental disorders, general practitioners were less likely to prescribe varenicline than NRT to patients with mental disorders compared to those without. There was little evidence consistent evidence that varenicline was associated with greater odds of depression, neurotic disorder, antidepressant, or hypnotic/anxiolytic prescription in patients with or without a mental health disorder. Our findings were broadly consistent across multivariable regression and propensity score matched models. However, the instrumental variable models were underpowered to detect treatment effect heterogeneity for most mental disorder subgroups, as the instrument only explains some of the variance in prescribed treatment.

### Strengths and Limitations

Data from the CPRD are representative of the UK population^30^ and other nations.^31^ Patients and clinicians were blinded to the study aims and therefore recall and reporting bias is unlikely to have influenced the results.

Exposure was routinely recorded in primary care,^30^ thus, unlikely to cause bias. The outcome, smoking status, is well reported in primary care,^32^ and was defined using expert-reviewed definitions, although it is unlikely that all patients had their smoking status consistently recorded. Analyses from our previous work showed that misclassification bias (ie, classing quitters as continuing smokers, vice versa) is unlikely to bias estimates of the effect of prescribing varenicline on cessation.^33^ Mental health diagnosis outcomes are less consistently recorded in the CPRD, however prescriptions of antidepressants and hypnotics/anxiolytics are routinely recorded. Where covariate data were missing we used multivariable multiple imputation to minimize selection bias.^28^

A strength of this study was the use of three different analytical methods to estimate the effectiveness and safety of varenicline.^34^ The propensity score analysis produced very similar findings to the multivariable regression. Both of these analyses are likely to be affected by residual confounding by unmeasured factors. For example, we are unable to account for tobacco addiction related characteristics (eg, nicotine dependence) which may affect both type of smoking cessation medication prescribed and likelihood of quitting smoking.

Our instrumental variable analyses used naturally occurring variation in the GPs’ prescribing to mimic randomisation to minimize bias due to patient characteristics. The instrumental variable estimates suggested that patients with mental disorders who were prescribed varenicline were more likely to have quit at 2 years. For every 100 smokers treated with varenicline about seven additional smokers would quit as compared to 100 smokers treated with NRT, and this association persisted up to 4 years. However, the estimates from the instrumental variable analyses were imprecise to examine some associations when stratified by mental disorder (ie, IV methods require large sample sizes, as IV regressions are less powered than a standard regression model because the instrument only explains some of the variance in prescribed treatment). In addition, while our previous work has shown that use of prescribing preferences is likely to be less affected by confounding than actual prescriptions received^23,35^ it is possible that clustering of patients with similar characteristics within GP surgeries may influence prescribing preferences.

We had no information on whether patients adhered to their smoking cessation medication treatment. Evidence from studies of similar databases show that rates of smoking cessation medication prescribing in primary care are comparable to national dispensing rates.^32^ Other studies have found that users of NRT continue taking the medication less than half the time it is prescribed. In our data, there is no information about when or if patients took medicines they were prescribed. This means that we estimated the effect of prescribing smoking cessation medications allowing for real-world treatment adherence.

### Comparison with Other Studies

Our smoking prevalence estimates are comparable to studies using similar databases for patients with mental disorders up to 2011,^4,16,36^ and to studies of smoking prevalence in the general population up to 2015.^3^ Varenicline and NRT prescribing rates in smokers with and without mental disorders are comparable to those reported in the THIN database,^4,16^ and the decreasing trend is similar to that reported by a UK database of National Health Service prescribing records (https://openprescribing.net). There have been substantial changes in the United Kingdom health care system over recent years, and considerable pressure on funding. One possibility is that these declines are a result of funding pressures. However, we cannot say with certainty solely based on prescribing data. Interestingly, despite this fall in prescribing rates, smoking in the population has declined considerably. Another potential explanation is the rising popularity of e-cigarettes in the United Kingdom may have reduced smoking treatment consulting.^37^ It is possible that patients are using e-cigarettes rather than varenicline and NRT. However, we cannot assess this possibility because our data contain no information on e-cigarette use. This would be an interesting topic for future research.

The EAGLES RCT compared the effect of varenicline versus NRT on smoking cessation in patients with mental disorders at 9- to 24-weeks follow-up.^11^ The trial included patients with mood disorders, neurotic disorders, psychotic disorders, and personality disorders (*N* = 4116). They found that varenicline produced higher quit rates than NRT. The effect estimate reported in EAGLES was similar to the estimate derived from our study in patients with mental disorders at 6-months follow-up, and the estimates from our study are more precise.

Our study is the largest to date to examine the effects of varenicline on mental health outcomes, stratified by mental disorder. Our finding that varenicline is associated with better or similar mental health outcomes than NRT similar to findings from a meta-analyses of observational studies in the general population and in those with depression.^38^ In our study, we found some weak evidence of an association between varenicline and greater odds of depression in people with schizophrenia and in patients prescribed mood stabilizers. Although this is the largest study to investigate this association specifically in these mental health subgroups, these findings are not consistent with the EAGLES trial.^11^ Our findings are in line with Molero et al.’s study that reported an association between varenicline and greater odds of mood conditions in people with psychiatric conditions,^10^ however they did not stratify analyses by mental disorder. We report our findings cautiously as our data are observational and are at risk of confounding. In addition, we did not observe the same pattern of results in these subgroups for prescription of antidepressants, which are likely to be recorded more accurately than diagnoses. Furthermore, results from our IV analyses were imprecise in patients with schizophrenia.

## Conclusions and Implications

Patients with mental disorders were less likely to be prescribed varenicline than NRT. Triangulating evidence across three analytical approaches, varenicline was more effective than NRT for smoking cessation in patients with mental disorders and was generally associated with decreased or similar odds of depression or anxiety compared to NRT. We report these findings cautiously as our data are observational and are at risk of confounding.

## Funding

This research was supported by Global Research Awards for Nicotine Dependence (GRAND), an independently reviewed competitive grants program supported by Pfizer, to the University of Bristol. GTs salary was funded by the National Institute for Health Research (NIHR) Health Technology Assessment (HTA) programme (project number 14/49/94) during the conduct of this research. The funders had no role in study design, data collection, and analysis, decision to publish, or preparation of the article. GT is currently funded by Cancer Research UK Population Researcher Postdoctoral Fellowship award (C56067/A21330). KHT was funded by a National Institute for Health Research (NIHR) Postdoctoral Fellowship (PDF-2017-10-068) for this research project. TJ receives funding from the National Institute for Health Research Collaboration for Leadership in Applied Health Research and Care (NIHR CLAHRC) West. The views expressed are those of the authors and not necessarily those of the NHS, the NIHR or the Department of Health. The MRC Integrative Epidemiology Unit is supported by the Medical Research Council and the University of Bristol (MC_UU_12013/6, MC_UU_12013/9). The research described in this article was partially funded by the Medical Research Council (MR/N01006X/1). This study was supported by the NIHR Biomedical Research Centre at University Hospitals Bristol NHS Foundation Trust and the University of Bristol. The views expressed in this publication are those of the author(s) and not necessarily those of the NHS, the National Institute for Health Research or the Department of Health and Social Care. The study sponsor and funder did not influence study conceptualization, design, analysis, interpretation, write up of any other part of this study and its conduct.

## Declaration of Interests

*GT, ND, and AT report a grant from Global Research Awards for Nicotine Dependence (GRAND), an independent grant making body funded by Pfizer, for the work conducted in this study. TI, TJ, RM, DR, FW, and KT have nothing to disclose. Dr. MRM reports grants from Pfizer, and grants and nonfinancial support from Rusan, outside the submitted work.*

## Author’s Contributions

GT contributed to study design, data cleaning, data analysis, interpretation of results, and writing the article. TI contributed to data analysis, interpretation of results, and writing the article. ND, AT, and KT contributed to study conceptualization, study design, interpretation of results, data analysis, and writing the article. RM, MM, DR, and FW contributed to study conceptualization, study design, interpretation of results, and writing the article. TJ extracted the data and contributed to writing the manuscript. GT, AT, and ND had full access to all of the data in the study and takes responsibility for the integrity of the data and the accuracy of the data analysis.

## Supplementary Material

ntz072_suppl_Supplementary-Material-1

ntz072_suppl_Supplementary-Material-2
