# Supplementary material for: Prescribing Prevalence, Effectiveness, and Mental Health Safety of Smoking Cessation Medicines in Patients With Mental Disorders
Source: Nicotine Tob Res. 2019 Jul 10;22(1):48–57. doi: 10.1093/ntr/ntz072 (PMC7073926; doi:10.1093/ntr/ntz072)
Supplement: ntz072_suppl_Supplementary-Material-1 [file ntz072_suppl_supplementary-material-1.docx]

Supplementary material 1

**Methods, results for the association between varenicline and smoking cessation outcomes, and model adequacy checks**

**Table of contents**

[Methods: Statistical analysis 3](#_Toc852545)

[Smoking and prescribing prevalence calculations 3](#_Toc852546)

[Relative effectiveness of varenicline versus NRT 4](#_Toc852547)

[eTable 1. Percent (%) and proportion (N) of patients with other mental disorder diagnoses or prescriptions 4](#_Toc852548)

[Multivariate logistic regression model 4](#_Toc852549)

[Propensity score matched logistic regression model 4](#_Toc852550)

[Instrumental variable analysis 5](#_Toc852551)

[Results 6](#_Toc852552)

[eFigure 1. Age and sex standardised percentage (%) of primary care patients with an electronic medical record indicating smoking, from 2006 to 2015, in patients with mental disorders, prescribed psychoactive medications, or with no mental disorder/psychoactive prescription. 7](#_Toc852553)

[eFigure 2. Age and sex standardised percentage (%) of primary care patients with an electronic medical record indicating smoking from 2006 to 2015, by mental disorder/psychoactive prescription 8](#_Toc852554)

[eFigure 3. Flow chart of the number (n) of patients and records assessed for eligibility and reasons for exclusion 9](#_Toc852555)

[eTable 2. Imputed baseline data for BMI and IMD* 10](#_Toc852556)

[eTable 3. Number and percentage (%) of smokers prescribed smoking cessation medications from 2006 to 2015, by mental disorder 11](#_Toc852557)

[eTable 4. The likelihood of smokers with mental disorders being prescribed varenicline versus NRT, as compared to smokers with no mental disorder, N=235,314 12](#_Toc852558)

[eTable 5. List of NRT products prescribed in study cohort 13](#_Toc852559)

[eTable 6. Number and percentage (%) of patients with an electronic medical record indicating smoking cessation at 3, 6 and 9-months, and 1, 2, and 4-years follow-up by exposure group, and by mental disorder 16](#_Toc852560)

[eTable 7. Stratified by mental disorder: Logistic regression partially adjusted odds ratios and 95% confidence intervals for the association between prescription of varenicline versus NRT and smoking cessation at 3, 6 and 9-months, and 1, 2, and 4-years after prescription 17](#_Toc852561)

[eTable 8 Stratified by mental disorder: Fully adjusted odds ratios and 95% confidence intervals for the association between prescription of varenicline versus NRT and smoking cessation at 3, 6 and 9-months and 1, 2, and 4-years after prescription 18](#_Toc852562)

[eTable 9. Bland-Altman tests for a difference in multivariable logistic regression coefficients for an association between varenicline and smoking cessation at 2-years follow-up: The difference between fully adjusted‡‡ odds ratios and 95% intervals derived from smokers with mental disorders, as compared to smokers with no mental disorder* 19](#_Toc852563)

[eTable 10. Stratified by mental disorder: Propensity score matched logistic regression odds ratios and 95% confidence intervals for the association between prescription of varenicline versus NRT and smoking cessation at 3, 6 and 9-months, and 1, 2, and 4-years follow-up 20](#_Toc852564)

[eTable 11. Stratified by mental disorder: Linear regression risk difference per 100 patients and 95% confidence intervals for the association between prescription of varenicline versus NRT and smoking cessation at 3, 6 and 9-months, and 1, 2, and 4-years follow-up 21](#_Toc852565)

[eTable 12. Stratified by mental disorder: Instrumental variable regression risk difference per 100 patients and 95% confidence intervals for the association between prescription of varenicline versus NRT and smoking cessation at 3, 6 and 9-months, and 1, 2, and 4-years follow-up 22](#_Toc852566)

[eTable 13. Bland-Altman tests for a difference in multivariable instrumental variable regression coefficients for an association between varenicline and smoking cessation at 2-years follow-up: The difference between adjusted‡ risk differences and 95% intervals derived from patients with mental disorders, compared to smokers with no mental disorder* 23](#_Toc852567)

[eFigure 4. Bias plot: Standardised % bias across covariates before and after propensity score matching 24](#_Toc852568)

[eFigure 5. Bias plot: Kernel density plots of propensity scores before and after matching 25](#_Toc852569)

[eFigure 6. Bias plot: Relative bias of linear regression and instrumental variable methods: binary and continuous outcomes 26](#_Toc852570)

[References 27](#_Toc852571)

# Methods: Statistical analysis

## Smoking and prescribing prevalence calculations

To calculate smoking and prescribing prevalence in patients with and without mental disorders we used the following formulae, and these were calculated for each year from 2006 to 2015:

1. The proportion of individuals with a mental health disorder who smoke:

$$\frac{\text{N smokers with mental disorder }}{\text{(N people with mental disorder}\text{ }-\text{N with mental health disorder and missing smoking data)}}$$

2. The proportion of individuals without a mental health disorder who smoke:

$$\frac{(N smokers) - (N smokers with mental disorder)}{\left( Total CPRD population-N missing smoking data \right)-(N with mental disorder-N with mental health disorder and missing smoking data)}$$

3. The proportion of smokers with a mental disorder prescribed smoking treatment:

$$\frac{N with mental disorder who were prescribed smoking treatment}{N smokers with a mental disorder}$$

1. The proportion of smokers without a mental disorder prescribed smoking treatment:

$$\frac{\left( N prescribed smoking treatment \right)-(N with mental disorder prescribed smoking treatment)}{(N smokers) -(N smokers with mental disorder)}$$

## Relative effectiveness of varenicline versus NRT

NB. In our protocol we pre-specified that we would analyse data for patients with eating disorders, personality disorders, hyperkinetic disorders, learning disabilities, autism, other behavioural/emotional conditions in childhood, and dementia, however the number of patients with these conditions in our data were too small to conduct effectiveness analyses. The proportion of patients with these disorders ever before prescription of smoking cessation medication is presented in eTable 1.

## eTable 1. Percent (%) and proportion (N) of patients with other mental disorder diagnoses or prescriptions

| **Mental disorder/prescription** | **Diagnosis/prescription ever before smoking cessation medication prescription** |
| --- | --- |
| Dementia | <1% (N= 435/235,314) |
| Eating disorder | 1.1% (N= 2,575/235,314) |
| Hyperkinetic disorders | <1% (N= 675/235,314) |
| Learning disorders | <1% (N= 1,531/235,314) |
| Childhood behavioural disorders | 1.8% (N= 4,169/235,314) |
| Personality disorders | 1.5% (N= 3,513/235,314) |
| Central nervous system medication | <1% (N= 544/235,314) |
| Dementia medication | <1% (N= 96/235,314) |

### Multivariate logistic regression model

We conducted a multivariate logistic regression model to estimate the association between prescription of varenicline versus NRT and smoking cessation/mental health outcomes at 3, 6 and 9-months and 1, 2, and 4-years after first prescription. Models were partially adjusted for age, sex, year of first prescription, and repeated with full adjustment for all baseline covariates. Models were repeated by mental disorder subgroup, and in smokers with no mental disorder/psychoactive prescription.

### Propensity score matched logistic regression model

Propensity score matching was used to create a sample of patients balanced on baseline covariates.(1–4) A propensity score matched model was created for each mental disorder (i.e. bipolar, depression, neurotic, stress-related and somatoform disorders, schizophrenia; or if they were prescribed antidepressants, antipsychotics, hypnotics or anxiolytics or mood stabilizers). Each participant’s propensity score was their conditional probability of receiving varenicline versus NRT based upon their baseline characteristics. Developing the propensity score model involved two steps: First using the psmatch2 command(1) a logistic regression model was estimated including all baseline covariates to calculate each participant’s propensity score. Within each mental disorder group each patient prescribed varenicline was matched to another patient prescribed NRT with the closest propensity score on a ratio of 1:1 using a nearest neighbor greedy algorithm, with no replacements, and matching was restricted to the common support region.(5–7) A logistic regression model was conducted in the matched sample with adjustment for propensity score to examine the association between prescription of smoking cessation medication and smoking cessation/mental health outcomes at all follow-up periods. Reporting of propensity score matching results followed reporting guidelines suggested by Thoemmes and Kim, as were model adequacy checks for balance and propensity score distributions.(8)

Model adequacy checks: All model adequacy checks were conducted using the matched sample of patients with any mental disorder. Firstly, the propensity score model was checked to ensure that a balance of means and variances was achieved for covariates after matching using the pstest command.(1) The standardized bias before and after matching was calculated to assess bias reduction. The standardized percent bias is the difference of the sample means in the NRT and varenicline groups (whole or matched sample), as a percentage of the square root of the average of the sample variances in each group.(1) Secondly, matching was restricted to within the common support region. This can be examined diagrammatically; plots of Kernel density estimations of groups’ propensity scores were examined by group, before and after matching.(8) If overlap between the group’s propensity score distributions is broad, this allows for causal estimates over the full range of propensity scores in the sample.(9) However, a small common support region restricts estimation of a causal effect and can result in bias by changing the observed population.(9)

### Instrumental variable analysis

We conducted an instrumental variable analysis to estimate the effectiveness of varenicline versus NRT on smoking cessation/mental health outcomes. Prescribing preferences for the prescriptions that GPs issued to their patients were used as the instrument. We averaged the physicians prescribing preference across their previous seven prescriptions of varenicline to increase power. Our instrument is much less strongly associated with potential confounders than traditional approaches and we have assessed this in bias plots in the Appendix 1, e-figure 6. This method has been adopted from the econometrics field and developed for use in epidemiology as a causal method.(7,10–15) Additive structural mean models estimated via the generalized method of moments were used to calculate risk differences in rates of cessation.(11,16,17) Smoking status was analyzed at 3, 6 and 9-months, and 1, 2, and 4-years after first prescription. A conventional linear regression was conducted to compare with the instrumental variable regression, and both approaches were conducted using the same sample. Models were repeated in smokers with mental disorders, and smokers with no mental disorder/psychoactive prescription.

# Results

## eFigure 1. Age and sex standardised percentage (%) of primary care patients with an electronic medical record indicating smoking, from 2006 to 2015, in patients with mental disorders, prescribed psychoactive medications, or with no mental disorder/psychoactive prescription.

## eFigure 2. Age and sex standardised percentage (%) of primary care patients with an electronic medical record indicating smoking from 2006 to 2015, by mental disorder/psychoactive prescription

eFigure 3. Flow chart of the number (n) of patients and records assessed for eligibility and reasons for exclusion

| Excluded from analysis due to protocol restrictions (Davies et al. 2015) | | |
| --- | --- | --- |
|  | Prescriptions | Patients |
| Prescriptions issued to patients under the age of 15 | 6,580 | 1,944 |
| Prescription issued after patients’ registration period ended | 0 | 0 |
| Prescription issued before patients’ registration period began | 296,490 | 53,529 |
| Prescription issued to patient aged 16 or 17 | 13,154 | 4,425 |
| Prescription issued before 1st September 2006 | 822,244 | 145,382 |
| Both varenicline and NRT were prescribed on the same day | 8,289 | 264 |
| Smoking cessation medication was not prescribed by a general practitioner (GP) | 141,810 | 16,168 |
| Smoking cessation medication prescription had less than one year of historical follow-up data prior to prescription | 163,821 | 41,112 |
| Bupropion prescriptions | 32,997 | 6,981 |
| Prescriptions issued to patients who received a smoking cessation medication issued in the previous 18 months | 1,069,188 | 30,278 |
| Patient had previously received an eligible smoking cessation medication prescription | 55,870 | 0 |
| Prescribing GP had seen less than 10 patients. | 18,494 | 18,494 |
| Patients had less than 180 days of follow-up after 1^st^ prescription. | 13,045 | 13,045 |
|  |  |  |
| Total excluded N= | 2,641,982 | 331,622 |

Assessed for eligibility

N Records=2,877,296

N Patients=566,936

Included in analysis

N patients=235,314

NRT

N patients=159,736

Varenicline

N patients=75,578

## eTable 2. Imputed baseline data for BMI and IMD*

| **Characteristic** | **Whole cohort** | **NRT** | **Varenicline** |
| --- | --- | --- | --- |
| BMI^1^ | 26.4 (0.1) | 26.4 (<0.01) | 26.4 (<0.01) |
| Multiple deprivation score (IMD)^2^ | 12 | 12 | 12 |
| *BMI data was missing for 14.1% (N= 33,059); IMD data was missing for 46.7% (N= 109,994). 1 Data presented are mean and standard error. 2 Data presented are median. | | | |

eTable 2 shows that there were no differences between the imputed, and non-imputed IMD, and BMI data.

## eTable 3. Number and percentage (%) of smokers prescribed smoking cessation medications from 2006 to 2015, by mental disorder

|  | **NRT**  **N (%)** | **Varenicline**  **N (%)** |
| --- | --- | --- |
| No mental disorder (N=156,857) | 100,396 (64.0%) | 56,461 (36.0%) |
| Any mental disorder (N=78,457) | 59,340 (75.6%) | 19,117 (24.4%) |
| Bipolar (N=2,012) | 1,799 (89.4%) | 213 (10.6%) |
| Depression (N=17,168) | 13,421 (78.2%) | 3,747(21.8%) |
| Neurotic disorder (N=4,704) | 4,263 (90.6%) | 441 (9.4%) |
| Schizophrenia (N=8,394) | 6,453(76.9%) | 1,941 (23.1%) |
| Antidepressants (N=56,756) | 43,589 (76.8%) | 13,167 (23.2%) |
| Antipsychotics (N=11,829) | 9,843 (83.2%) | 1,986 (16.8%) |
| Hypnotics/anxiolytics (N=31,291) | 23,651 (75.6%) | 7,640 (24.4%) |
| Mood stabilisers (N=4,728) | 4,079 (86.3%) | 649 (13.7%) |

eTable 3 shows the number and percentage of smokers with mental disorders who were prescribed varenicline and NRT during the study period. Smokers with no mental disorder were more commonly prescribed varenicline than were smokers with mental disorders.

## eTable 4. The likelihood of smokers with mental disorders being prescribed varenicline versus NRT, as compared to smokers with no mental disorder, N=235,314

| **Logistic regression models**  **Odds ratio (95% confidence interval)** | | |
| --- | --- | --- |
|  | **Partially adjusted†** | **Fully adjusted††** |
| Any mental disorder | 0.57 (0.56 to 0.58) | 0.69 (0.67 to 0.71) |
| Bipolar | 0.24 (0.21 to 0.28) | 0.56 (0.48 to 0.65) |
| Depression | 0.57 (0.55 to 0.59) | 0.7 (0.67 to 0.73) |
| Neurotic disorder | 0.63 (0.59 to 0.66) | 0.8 (0.75 to 0.84) |
| Schizophrenia | 0.20 (0.18 to 0.22) | 0.3 (0.27 to 0.34) |
| Antidepressants | 0.55 (0.54 to 0.56) | 0.64 (0.62 to 0.66) |
| Antipsychotics | 0.41 (0.39 to 0.43) | 0.71 (0.67 to 0.76) |
| Hypnotics/anxiolytics | 0.67 (0.65 to 0.69) | 0.84 (0.82 to 0.87) |
| Mood stabilisers | 0.33 (0.30 to 0.36) | 0.57 (0.51 to 0.64) |
| ‡Partially adjusted models were adjusted for: age, sex, year of first prescription. ††Fully adjusted models adjusted for all baseline covariates. All models were estimated using cluster robust standard errors to account for potential clustering of patients between practices. | | |

eTable 4 presents partially and fully adjusted odds ratios and 95% confidence intervals for likelihood of those with mental disorders being prescribed varenicline versus NRT, as compared to smokers with no mental disorder. Smokers with mental disorders were less likely to be prescribed varenicline compared to smokers with no mental disorder.

## eTable 5. List of NRT products prescribed in study cohort

| **Prodcode** | **Product** **name** |
| --- | --- |
| 41778 | Nicorette Fruitfusion 4mg medicated chewing gum (McNeil Products Ltd) |
| 40617 | Nicotinell TTS 20 patches (Novartis Consumer Health UK Ltd) |
| 5320 | Nicorette 10mg Inhalator (McNeil Products Ltd) |
| 45429 | Nicorette QuickMist 1mg/dose mouthspray (McNeil Products Ltd) |
| 1703 | Nicorette 15mg Transdermal patch (Pharmacia Ltd) |
| 41372 | NiQuitin Clear 21mg patches (GlaxoSmithKline Consumer Healthcare) |
| 46592 | Nicorette 15mg Inhalator (McNeil Products Ltd) |
| 6448 | Nicotine 21mg/24hours transdermal patches |
| 39166 | Nicorette invisi 15mg/16hours patches (McNeil Products Ltd) |
| 38958 | Nicotinell 1mg lozenges (Novartis Consumer Health UK Ltd) |
| 5479 | Nicotine 10mg/16hours transdermal patches |
| 5944 | Nicotine 10mg inhalation cartridges with device |
| 5502 | Nicotine 15mg/16hours transdermal patches |
| 45504 | Nicotine 1mg/dose oromucosal spray sugar free |
| 3404 | Niquitin 21mg Transdermal patch (GlaxoSmithKline Consumer Healthcare) |
| 6565 | NiQuitin Mint 2mg medicated chewing gum (GlaxoSmithKline Consumer Healthcare) |
| 5758 | Nicotine 4mg medicated chewing gum sugar free |
| 9806 | Nicotine 2mg lozenges sugar free |
| 40730 | NiQuitin Minis Mint 1.5mg lozenges (GlaxoSmithKline Consumer Healthcare) |
| 2876 | Nicorette Citrus 2mg medicated chewing gum (Pfizer Ltd) |
| 41368 | NiQuitin 21mg patches (GlaxoSmithKline Consumer Healthcare) |
| 41802 | Nicorette 5mg patches (McNeil Products Ltd) |
| 39046 | Nicorette invisi 25mg/16hours patches (McNeil Products Ltd) |
| 4717 | Niquitin 14mg Transdermal patch (GlaxoSmithKline Consumer Healthcare) |
| 4166 | Nicorette Citrus 4mg medicated chewing gum (Pfizer Ltd) |
| 41505 | NiQuitin Clear 14mg patches (GlaxoSmithKline Consumer Healthcare) |
| 9804 | Nicotine 7mg/24hours transdermal patches |
| 9591 | Nicotine 14mg/24hours transdermal patches |
| 4704 | Niquitin 7mg Transdermal patch (GlaxoSmithKline Consumer Healthcare) |
| 41753 | Nicorette Original 4mg medicated chewing gum (McNeil Products Ltd) |
| 6698 | Nicotinell 2mg lozenges (Novartis Consumer Health UK Ltd) |
| 1248 | Nicorette 10mg/ml Nasal spray (Pharmacia Ltd) |
| 41474 | Nicorette 10mg patches (McNeil Products Ltd) |
| 5515 | Nicotine 1mg Lozenge |
| 5606 | Nicotinell tts 20 sq cm Transdermal patch (Novartis Consumer Health UK Ltd) |
| 11718 | Nicotine 2mg sublingual tablets sugar free |
| 41493 | Nicorette Icy White 4mg medicated chewing gum (McNeil Products Ltd) |
| 40620 | Nicotinell TTS 30 patches (Novartis Consumer Health UK Ltd) |
| 7303 | Nicotinell tts 10 sq cm Transdermal patch (Novartis Consumer Health UK Ltd) |
| 41356 | Nicorette Microtab 2mg sublingual tablets (McNeil Products Ltd) |
| 46717 | Nicotine 15mg inhalation cartridges with device |
| 5946 | Nicotinell 2mg Medicated chewing-gum (Novartis Consumer Health UK Ltd) |
| 36457 | Nicopatch 21mg/24hours transdermal patches (Pierre Fabre Ltd) |
| 6323 | Nicotine 2mg medicated chewing gum sugar free |
| 41765 | Nicotinell Mint 2mg medicated chewing gum (Novartis Consumer Health UK Ltd) |
| 41425 | Nicorette Freshmint 4mg medicated chewing gum (McNeil Products Ltd) |
| 3818 | Nicotinell tts 30 sq cm Transdermal patch (Novartis Consumer Health UK Ltd) |
| 37646 | Nicotine 1.5mg lozenges sugar free |
| 41376 | Nicorette 15mg patches (McNeil Products Ltd) |
| 49319 | Nicorette Cools 4mg lozenges (McNeil Products Ltd) |
| 41485 | NiQuitin 14mg patches (GlaxoSmithKline Consumer Healthcare) |
| 5440 | Nicorette 10mg Transdermal patch (Pharmacia Ltd) |
| 41507 | NiQuitin Clear 7mg patches (GlaxoSmithKline Consumer Healthcare) |
| 39123 | Nicotine 25mg/16hours transdermal patches |
| 40865 | NiQuitin Minis Mint 4mg lozenges (GlaxoSmithKline Consumer Healthcare) |
| 5457 | Nicotine 5mg/16hours transdermal patches |
| 5659 | NiQuitin 4mg lozenges original menthol mint (GlaxoSmithKline Consumer Healthcare) |
| 5700 | NiQuitin 2mg lozenges original menthol mint (GlaxoSmithKline Consumer Healthcare) |
| 41864 | Nicorette Freshfruit 2mg medicated chewing gum (McNeil Products Ltd) |
| 5877 | Nicorette 2mg microtab (Pharmacia Ltd) |
| 6018 | Nicorette 5mg Transdermal patch (Pharmacia Ltd) |
| 41779 | Nicorette Icy White 2mg medicated chewing gum (McNeil Products Ltd) |
| 13048 | Nicotinell 4mg Medicated chewing-gum (Novartis Consumer Health UK Ltd) |
| 5784 | Nicotine 4mg lozenges sugar free |
| 41496 | Nicorette 500micrograms/dose nasal spray (McNeil Products Ltd) |
| 49305 | Nicorette Cools 2mg lozenges (McNeil Products Ltd) |
| 41426 | NiQuitin 7mg patches (GlaxoSmithKline Consumer Healthcare) |
| 6642 | NiQuitin Mint 4mg medicated chewing gum (GlaxoSmithKline Consumer Healthcare) |
| 39572 | Nicorette invisi 10mg/16hours patches (McNeil Products Ltd) |
| 36635 | Nicopatch 14mg/24hours transdermal patches (Pierre Fabre Ltd) |
| 44106 | NiQuitin Minis Cherry 1.5mg lozenges (GlaxoSmithKline Consumer Healthcare) |
| 8571 | Nicotine 500micrograms/dose nasal spray |
| 57829 | NiQuitin Strips Mint 2.5mg oral films (GlaxoSmithKline Consumer Healthcare) |
| 5531 | Nicotinell 1mg Lozenge (Novartis Consumer Health UK Ltd) |
| 41377 | Nicorette Original 2mg medicated chewing gum (McNeil Products Ltd) |
| 41809 | Nicorette Mint 4mg medicated chewing gum (McNeil Products Ltd) |
| 40683 | Nicotinell TTS 10 patches (Novartis Consumer Health UK Ltd) |
| 6630 | NiQuitin Mint 2mg lozenges (GlaxoSmithKline Consumer Healthcare) |
| 45603 | Nicorette Freshmint 2mg lozenges (McNeil Products Ltd) |
| 46588 | Nicotinell Icemint 2mg medicated chewing gum (Novartis Consumer Health UK Ltd) |
| 41909 | Nicotinell Mint 4mg medicated chewing gum (Novartis Consumer Health UK Ltd) |
| 42011 | Nicotinell Classic 4mg medicated chewing gum (Novartis Consumer Health UK Ltd) |
| 6593 | NiQuitin Mint 4mg lozenges (GlaxoSmithKline Consumer Healthcare) |
| 46701 | Nicotinell Icemint 4mg medicated chewing gum (Novartis Consumer Health UK Ltd) |
| 41808 | Nicotinell Fruit 4mg medicated chewing gum (Novartis Consumer Health UK Ltd) |
| 42048 | Nicotine bitartrate 1mg lozenges sugar free |
| 41801 | Nicorette Freshmint 2mg medicated chewing gum (McNeil Products Ltd) |
| 41881 | Nicotinell Classic 2mg medicated chewing gum (Novartis Consumer Health UK Ltd) |
| 58034 | Nicotine 2.5mg orodispersible films sugar free |
| 54102 | NiQuitin Pre-Quit Clear 21mg patches (GlaxoSmithKline Consumer Healthcare) |
| 58410 | NiQuitin Minis Orange 1.5mg lozenges (GlaxoSmithKline Consumer Healthcare) |
| 48620 | Boots NicAssist 10mg Inhalator (The Boots Company Plc) |
| 36618 | Nicopatch 7mg/24hours transdermal patches (Pierre Fabre Ltd) |
| 41931 | Nicotinell Fruit 2mg medicated chewing gum (Novartis Consumer Health UK Ltd) |
| 37716 | Nicopass 1.5mg Lozenge (Wockhardt UK Ltd) |
| 42016 | Nicorette Mint 2mg medicated chewing gum (McNeil Products Ltd) |
| 42286 | Nicotine bitartrate 2mg lozenges sugar free |
| 41040 | Nicorette lemon 2mg microtab (McNeil Products Ltd) |
| 42047 | Nicotinell Liquorice 4mg medicated chewing gum (Novartis Consumer Health UK Ltd) |
| 41923 | Nicotine 15mg/16 hours transdermal patches and Nicotine 2mg medicated chewing gum sugar free |
| 42400 | Nicorette combi 15mg transdermal patches + 2mg medicated gum (McNeil Products Ltd) |
| 39521 | NiQuitin Pre-Quit Mint 4mg lozenges (GlaxoSmithKline Consumer Healthcare) |
| 25510 | Nicotine 2mg mint flavour chewing-gum |
| 41879 | Nicotinell Liquorice 2mg medicated chewing gum (Novartis Consumer Health UK Ltd) |

## eTable 6. Number and percentage (%) of patients with an electronic medical record indicating smoking cessation at 3, 6 and 9-months, and 1, 2, and 4-years follow-up by exposure group, and by mental disorder

|  | **Treatment** | **3-months** | **6-months** | **9-months** | **1-year** | **2-years** | **4-years** |
| --- | --- | --- | --- | --- | --- | --- | --- |
| No mental disorder | NRT (N=100,396) | 14,669 (14.6%) | 18,504 (18.4%) | 20,490 (20.4%) | 21,947 (21.9%) | 25,270 (25.2%) | 29,904 (29.8%) |
|  | Varenicline (N=56,461) | 10,683 (18.9%) | 13,448 (23.8%) | 14,486 (25.7%) | 15,133 (26.8%) | 16,655 (29.5%) | 18,832 (33.4%) |
| Any mental disorder | NRT (N=59,340) | 7,544 (12.7%) | 9,694 (16.3%) | 10,836 (18.3%) | 11,661 (19.7%) | 13,426 (22.6%) | 15,599 (26.3%) |
|  | Varenicline (N=19,117) | 3,378 (17.7%) | 4,227 (22.1%) | 4,473 (23.4%) | 4,624 (24.2%) | 5,043 (26.4%) | 5,662 (29.6%) |
| Bipolar | NRT (N=1,799) | 223 (12.4%) | 281 (15.6%) | 317 (17.6%) | 338 (18.8%) | 390 (21.7%) | 481 (26.7%) |
|  | Varenicline (N=213) | 36 (16.9%) | 52 (24.4%) | 52 (24.4%) | 48 (22.5%) | 55 (25.8%) | 63 (29.6%) |
| Depression | NRT (N=13,421) | 1,544 (11.5%) | 1,945 (14.5%) | 2,152 (16%) | 2,303 (17.2%) | 2,659 (19.8%) | 3,195 (23.8%) |
|  | Varenicline (N=3,747) | 611 (16.3%) | 754 (20.1%) | 785 (21%) | 833 (22.2%) | 878 (23.4%) | 976 (26%) |
| Neurotic disorder | NRT (N=6,453) | 786 (12.2%) | 1,030 (16%) | 1,129 (17.5%) | 1,232 (19.1%) | 1,358 (21%) | 1,625 (25.2%) |
|  | Varenicline (N=1,941) | 328 (16.9%) | 403 (20.8%) | 433 (22.3%) | 456 (23.5%) | 496 (25.6%) | 562 (29%) |
| Schizophrenia | NRT (N=4,263) | 478 (11.2%) | 589 (13.8%) | 669 (15.7%) | 716 (16.8%) | 817 (19.2%) | 942 (22.1%) |
|  | Varenicline (N=441) | 72 (16.3%) | 88 (20%) | 96 (21.8%) | 95 (21.5%) | 109 (24.7%) | 107 (24.3%) |
| Antidepressants | NRT (N=43,589) | 5,479 (12.6%) | 7,020 (16.1%) | 9,735 (22.3%) | 8,421 (19.3%) | 9,735 (22.3%) | 11,334 (26%) |
|  | Varenicline (N=13,167) | 2,283 (17.3%) | 2,860 (21.7%) | 3,388 (25.7%) | 3,110 (23.6%) | 3,388 (25.7%) | 3,796 (28.8%) |
| Antipsychotics | NRT (N=9,843) | 1,221 (12.4%) | 1,580 (16.1%) | 1,752 (17.8%) | 1,888 (19.2%) | 2,169 (22%) | 2,496 (25.4%) |
|  | Varenicline (N=1,986) | 348 (17.5%) | 441 (22.2%) | 464 (23.4%) | 456 (23%) | 521 (26.2%) | 583 (29.4%) |
| Hypnotics/anxiolytics | NRT (N=23,651) | 2,883 (12.2%) | 3,690 (15.6%) | 4,124 (17.4%) | 4,475 (18.9%) | 5,197 (22%) | 5,912 (25%) |
|  | Varenicline (N=7,640) | 1,288 (16.9%) | 1,634 (21.4%) | 1,736 (22.7%) | 1,798 (23.5%) | 1,972 (25.8%) | 2,222 (29.1%) |
| Mood stabilisers | NRT (N=4,079) | 505 (12.4%) | 654 (16%) | 728 (17.8%) | 787 (19.3%) | 891 (21.8%) | 1,009 (24.7%) |
|  | Varenicline (N=649) | 105 (16.2%) | 140 (21.6%) | 163 (25.1%) | 164 (25.3%) | 183 (28.2%) | 201 (31.0%) |

eTable 6 shows that the proportion of quitters was higher at all follow-ups in patients prescribed varenicline compared to NRT for all mental disorders, except for in smokers with schizophrenia NRT produced higher quit rates at 9-months, 1 and 4-years.

## eTable 7. Stratified by mental disorder: Logistic regression partially adjusted odds ratios and 95% confidence intervals for the association between prescription of varenicline versus NRT and smoking cessation at 3, 6 and 9-months, and 1, 2, and 4-years after prescription

| **Logistic regression models**  **Partially adjusted odds ratio (95% confidence interval) †** | | | | | | |
| --- | --- | --- | --- | --- | --- | --- |
|  | **3-months** | **6-months** | **9-months** | **1-year** | **2-years** | **4-years** |
| No mental disorder (N=156,857) | 1.42 (1.37 to 1.47) | 1.46 (1.42 to 1.51) | 1.42 (1.37 to 1.46) | 1.37 (1.33 to 1.41) | 1.28 (1.25 to 1.32) | 1.20 (1.17 to 1.23) |
| Any mental disorder (N=78,457) | 1.51 (1.44 to 1.59) | 1.50 (1.43 to 1.56) | 1.40 (1.34 to 1.47) | 1.33 (1.27 to 1.38) | 1.24 (1.19 to 1.29) | 1.18 (1.14 to 1.23) |
| Bipolar (N=2,011) | 1.42 (0.96 to 2.10) | 1.76 (1.25 to 2.47) | 1.55 (1.11 to 2.17) | 1.28 (0.90 to 1.80) | 1.26 (0.91 to 1.75) | 1.17 (0.85 to 1.60) |
| Depression (N=17,168) | 1.50 (1.35 to 1.66) | 1.48 (1.34 to 1.63) | 1.38 (1.25 to 1.51) | 1.36 (1.24 to 1.50) | 1.22 (1.12 to 1.33) | 1.11 (1.02 to 1.21) |
| Neurotic disorder (N=8,394) | 1.49 (1.29 to 1.73) | 1.39 (1.22 to 1.60) | 1.36 (1.20 to 1.55) | 1.31 (1.15 to 1.49) | 1.29 (1.15 to 1.46) | 1.21 (1.08 to 1.35) |
| Schizophrenia (N=4,704) | 1.53 (1.15 to 2.04) | 1.55 (1.19 to 2.02) | 1.51 (1.17 to 1.94) | 1.35 (1.05 to 1.74) | 1.38 (1.09 to 1.75) | 1.13 (0.90 to 1.42) |
| Antidepressants (N=56,756) | 1.49 (1.41 to 1.58) | 1.48 (1.40 to 1.56) | 1.38 (1.31 to 1.45) | 1.30 (1.24 to 1.37) | 1.21 (1.15 to 1.26) | 1.15 (1.10 to 1.20) |
| Antipsychotics (N=11,829) | 1.52 (1.33 to 1.75) | 1.51 (1.33 to 1.71) | 1.43 (1.27 to 1.62) | 1.27 (1.13 to 1.43) | 1.27 (1.13 to 1.43) | 1.23 (1.10 to 1.38) |
| Hypnotics/ anxiolytics (N=31,291) | 1.52 (1.41 to 1.64) | 1.53 (1.43 to 1.64) | 1.44 (1.35 to 1.55) | 1.36 (1.27 to 1.45) | 1.27 (1.19 to 1.35) | 1.26 (1.18 to 1.33) |
| Mood stabilisers (N=4,727) | 1.37 (1.08 to 1.73) | 1.45 (1.16 to 1.80) | 1.57 (1.28 to 1.93) | 1.41 (1.15 to 1.73) | 1.40 (1.15 to 1.70) | 1.35 (1.12 to 1.63) |
| ‡ Partially adjusted models were adjusted for: age, sex, year of first prescription. Models were estimated using cluster robust standard errors to account for potential clustering of patients between practices. | | | | | | |

eTable 7 presents partially adjusted odds ratios and 95% confidence intervals for the relative association between prescription of varenicline compared to NRT and smoking cessation, stratified by mental disorder. Varenicline was associated with higher odds of quitting smoking at all follow-ups in most mental disorders. There was some evidence that varenicline was no more effective than NRT in smokers with bipolar at 3-months, and at 1, 2, and 4-years follow-up.

## eTable 8 Stratified by mental disorder: Fully adjusted odds ratios and 95% confidence intervals for the association between prescription of varenicline versus NRT and smoking cessation at 3, 6 and 9-months and 1, 2, and 4-years after prescription

| **Logistic regression models**  **Fully adjusted odds ratio (95% confidence interval)** †† | | | | | | |
| --- | --- | --- | --- | --- | --- | --- |
|  | 3-months | 6-months | 9-months | 1-year | 2-years | 4-years |
| No mental disorder (N=156,857) | 1.40 (1.35 to 1.46) | 1.45 (1.41 to 1.50) | 1.40 (1.36 to 1.45) | 1.35 (1.31 to 1.39) | 1.26 (1.23 to 1.3) | 1.18 (1.15 to 1.21) |
| Any mental disorder (N=78,457) | 1.48 (1.40 to 1.55) | 1.45 (1.39 to 1.52) | 1.36 (1.31 to 1.42) | 1.29 (1.23 to 1.34) | 1.19 (1.15 to 1.24) | 1.14 (1.09 to 1.18) |
| Bipolar (N=2,011) | 1.38 (0.93 to 2.06) | 1.77 (1.25 to 2.49) | 1.50 (1.07 to 2.12) | 1.23 (0.86 to 1.75) | 1.22 (0.87 to 1.70) | 1.12 (0.81 to 1.54) |
| Depression (N=17,168) | 1.48 (1.33 to 1.64) | 1.46 (1.32 to 1.61) | 1.36 (1.23 to 1.49) | 1.35 (1.23 to 1.48) | 1.20 (1.10 to 1.31) | 1.09 (1.00 to 1.18) |
| Neurotic disorder (N=8,394) | 1.47 (1.27 to 1.70) | 1.38 (1.20 to 1.58) | 1.34 (1.17 to 1.53) | 1.29 (1.13 to 1.47) | 1.27 (1.12 to 1.43) | 1.17 (1.04 to 1.31) |
| Schizophrenia (N=4,704) | 1.53 (1.15 to 2.05) | 1.55 (1.18 to 2.02) | 1.50 (1.16 to 1.94) | 1.34 (1.04 to 1.73) | 1.33 (1.05 to 1.7) | 1.10 (0.87 to 1.39) |
| Antidepressants (N=56,756) | 1.46 (1.38 to 1.55) | 1.44 (1.37 to 1.52) | 1.35 (1.28 to 1.42) | 1.27 (1.21 to 1.33) | 1.17 (1.11 to 1.22) | 1.11 (1.06 to 1.16) |
| Antipsychotics (N=11,829) | 1.46 (1.26 to 1.68) | 1.42 (1.25 to 1.61) | 1.34 (1.19 to 1.52) | 1.17 (1.03 to 1.32) | 1.16 (1.03 to 1.31) | 1.13 (1.01 to 1.27) |
| Hypnotics/ anxiolytics (N=31,291) | 1.45 (1.34 to 1.56) | 1.46 (1.36 to 1.56) | 1.37 (1.28 to 1.46) | 1.28 (1.20 to 1.37) | 1.20 (1.12 to 1.27) | 1.18 (1.12 to 1.26) |
| Mood stabilisers (N=4,727) | 1.32 (1.04 to 1.68) | 1.41 (1.13 to 1.76) | 1.54 (1.25 to 1.89) | 1.37 (1.12 to 1.69) | 1.37 (1.13 to 1.68) | 1.33 (1.10 to 1.62) |
| ‡‡ Fully adjusted models were adjusted for: age, sex, days in history, IMD, number of GP visits 1-year prior to first prescription, BMI, year of first prescription, history of major physical morbidity (Charlson Index), alcohol misuse ever, drug misuse ever, bipolar ever, depression ever, neurotic disorder ever, self-harm ever, other mental disorder ever, antidepressant prescription ever, antipsychotic prescription ever, hypnotics/anxiolytics prescription ever, other psychoactive medication ever, and other behavioral/neurologic disorder ever. Missing BMI and IMD values were imputed using multiple imputation. | | | | | | |

eTable 8 presents partially adjusted odds ratios and 95% confidence intervals for the relative association between prescription of varenicline compared to NRT and smoking cessation, stratified by mental disorder. Varenicline was associated with higher odds of quitting smoking at all follow-ups in most mental disorders. There was some evidence that varenicline was no more effective than NRT in smokers with bipolar at 3-months, and at 1, 2, and 4-years follow-up, and in people with schizophrenia at 4-years.

## eTable 9. Bland-Altman tests for a difference in multivariable logistic regression coefficients for an association between varenicline and smoking cessation at 2-years follow-up: The difference between fully adjusted‡‡ odds ratios and 95% intervals derived from smokers with mental disorders, as compared to smokers with no mental disorder*

|  | **P-value for difference in coefficients** |
| --- | --- |
| Any mental disorder (N=78,457) | 0.02 |
| Bipolar (N=2,011) | 0.39 |
| Depression (N=17,168) | 0.21 |
| Neurotic disorder (N=4,704) | 0.40 |
| Schizophrenia (N=8,394) | 0.37 |
| Antidepressants (N=56,756) | 0.01 |
| Antipsychotics (N=11,829) | 0.15 |
| Hypnotics/anxiolytics (N=31,291) | 0.11 |
| Mood stabilisers (N=4,727) | 0.28 |
| ‡‡ Fully adjusted models were adjusted for: age, sex, days in history, IMD, number of GP visits 1-year prior to first prescription, BMI, year of first prescription, history of major physical morbidity (Charlson Index), alcohol misuse ever, drug misuse ever, bipolar ever, depression ever, neurotic disorder ever, self-harm ever, other mental disorder ever, antidepressant prescription ever, antipsychotic prescription ever, hypnotics/anxiolytics prescription ever, other psychotropic medication ever, other behavioural/neurologic disorder ever. Models were estimated using cluster robust standard errors to account for potential clustering of patients between practices. Missing BMI and IMD values were imputed using multiple imputation. *No mental disorder (N=156,857). | |

## eTable 10. Stratified by mental disorder: Propensity score matched logistic regression odds ratios and 95% confidence intervals for the association between prescription of varenicline versus NRT and smoking cessation at 3, 6 and 9-months, and 1, 2, and 4-years follow-up

| **Propensity score matched logistic regression models**  **Adjusted odds ratio (95% confidence interval) †*** | | | | | | |
| --- | --- | --- | --- | --- | --- | --- |
|  | **3-months** | **6-months** | **9-months** | **1-year** | **2-years** | **4-years** |
| No mental disorder (N=112,910) | 1.40 (1.34 to 1.46) | 1.43 (1.38 to 1.49) | 1.39 (1.34 to 1.44) | 1.34 (1.29 to 1.38) | 1.25 (1.22 to 1.29) | 1.19 (1.15 to 1.22) |
| Any mental disorder (N=38,222) | 1.45 (1.36 to 1.56) | 1.42 (1.34 to 1.52) | 1.34 (1.26 to 1.42) | 1.27 (1.20 to 1.35) | 1.18 (1.12 to 1.24) | 1.14 (1.08 to 1.20) |
| Bipolar (N=418) | 1.46 (0.75 to 2.81) | 1.81 (1.00 to 3.28) | 1.53 (0.85 to 2.75) | 1.26 (0.70 to 2.27) | 1.23 (0.72 to 2.12) | 1.11 (0.66 to 1.89) |
| Depression (N=7,484) | 1.45 (1.24 to 1.69) | 1.43 (1.24 to 1.64) | 1.33 (1.15 to 1.53) | 1.31 (1.14 to 1.51) | 1.18 (1.04 to 1.33) | 1.08 (0.96 to 1.21) |
| Neurotic disorder (N=3,874) | 1.50 (1.17 to 1.92) | 1.39 (1.11 to 1.74) | 1.36 (1.11 to 1.66) | 1.31 (1.08 to 1.59) | 1.29 (1.07 to 1.54) | 1.21 (1.02 to 1.43) |
| Schizophrenia (N=876) | 1.50 (0.95 to 2.38) | 1.51 (1.02 to 2.24) | 1.48 (0.98 to 2.22) | 1.32 (0.90 to 1.94) | 1.32 (0.90 to 1.95) | 1.07 (0.72 to 1.58) |
| Antidepressants (N=26,330) | 1.44 (1.32 to 1.56) | 1.41 (1.31 to 1.52) | 1.31 (1.22 to 1.41) | 1.25 (1.16 to 1.33) | 1.15 (1.07 to 1.23) | 1.11 (1.04 to 1.18) |
| Antipsychotics (N=3,970) | 1.46 (1.17 to 1.82) | 1.42 (1.18 to 1.70) | 1.33 (1.11 to 1.59) | 1.16 (0.98 to 1.37) | 1.14 (0.97 to 1.35) | 1.13 (0.96 to 1.32) |
| Hypnotics/anxiolytics (N=15,270) | 1.43 (1.28 to 1.59) | 1.43 (1.30 to 1.58) | 1.36 (1.23 to 1.49) | 1.28 (1.16 to 1.40) | 1.19 (1.09 to 1.30) | 1.19 (1.10 to 1.30) |
| Mood stabilisers (N=1,284) | 1.30 (0.89 to 1.91) | 1.36 (0.94 to 1.95) | 1.45 (1.03 to 2.06) | 1.34 (0.95 to 1.90) | 1.30 (0.95 to 1.78) | 1.32 (0.97 to 1.80) |
| ‡‡Estimates were adjusted for propensity score, and propensity score. Models were estimated using cluster robust standard errors to account for potential clustering of patients between practices. | | | | | | |

eTable 10 presents propensity score matched odds ratios and 95% confidence intervals for the relative association between prescription of varenicline compared to NRT, and smoking cessation, stratified by mental disorder. Varenicline was associated with higher odds of quitting smoking at all follow-ups in smokers with most mental disorders. There was some evidence that varenicline was no more effective than NRT in smokers with bipolar, or schizophrenia at all follow-ups except 6-months, and in those prescribed antipsychotics at 1, 2, and 4-years follow-up.

## eTable 11. Stratified by mental disorder: Linear regression risk difference per 100 patients and 95% confidence intervals for the association between prescription of varenicline versus NRT and smoking cessation at 3, 6 and 9-months, and 1, 2, and 4-years follow-up

| **Linear regression models**  **Risk difference per 100 patients (95% confidence interval) ‡‡** | | | | | | |
| --- | --- | --- | --- | --- | --- | --- |
|  | **3-months** | **6-months** | **9-months** | **1-year** | **2-years** | **4-years** |
| No mental disorder (N=136,654) | 4.38 (3.83 to 4.94) | 5.61 (5.02 to 6.20) | 5.37 (4.78 to 5.96) | 5.01 (4.43 to 5.58) | 4.18 (3.63 to 4.74) | 3.39 (2.82 to 3.96) |
| Any mental disorder (N=68,578) | 5.07 (4.36 to 5.78) | 5.77 (5.01 to 6.52) | 5.11 (4.36 to 5.87) | 4.50 (3.74 to 5.26) | 3.65 (2.91 to 4.40) | 3.08 (2.32 to 3.85) |
| Bipolar (N=1,735) | 4.28 (-1.17 to 9.74) | 9.19 (2.97 to 15.42) | 6.81 (0.56 to 13.06) | 3.21 (-2.79 to 9.21) | 2.97 (-3.30 to 9.24) | 3.75 (-2.97 to 10.47) |
| Depression (N=14,695) | 4.85 (3.46 to 6.24) | 5.47 (3.96 to 6.97) | 4.86 (3.33 to 6.40) | 5.20 (3.61 to 6.79) | 3.57 (2.00 to 5.14) | 2.40 (0.75 to 4.06) |
| Neurotic disorder (N=4,125) | 5.32 (3.30 to 7.33) | 5.20 (3.00 to 7.40) | 5.18 (2.91 to 7.44) | 4.93 (2.62 to 7.24) | 4.43 (2.13 to 6.72) | 3.00 (0.64 to 5.36) |
| Schizophrenia (N=7,269) | 5.64 (1.75 to 9.53) | 6.38 (2.16 to 10.60) | 6.24 (1.90 to 10.59) | 5.11 (0.77 to 9.45) | 6.05 (1.61 to 10.49) | 2.92 (-1.37 to 7.21) |
| Anti-depressants (N=49,763) | 4.92 (4.11 to 5.73) | 5.59 (4.72 to 6.46) | 4.90 (4.02 to 5.78) | 4.27 (3.38 to 5.16) | 3.18 (2.27 to 4.08) | 2.54 (1.62 to 3.47) |
| Anti-psychotics (N=10,345) | 5.03 (3.05 to 7.01) | 5.95 (3.84 to 8.06) | 5.23 (3.10 to 7.37) | 3.49 (1.38 to 5.60) | 3.74 (1.48 to 6.01) | 3.63 (1.30 to 5.96) |
| Hypnotics/anxiolytics (N=27,214) | 4.71 (3.69 to 5.74) | 5.64 (4.52 to 6.76) | 4.97 (3.82 to 6.11) | 4.36 (3.21 to 5.52) | 3.69 (2.52 to 4.86) | 3.67 (2.45 to 4.89) |
| Mood stabilisers (N=4,104) | 4.21 (0.98 to 7.45) | 6.15 (2.42 to 9.89) | 8.03 (4.16 to 11.9) | 6.72 (2.82 to 10.63) | 6.53 (2.55 to 10.52) | 6.57 (2.43 to 10.71) |
| ‡‡Partially adjusted estimates were adjusted for age, sex, year of prescription. Models were estimated using cluster robust standard errors to account for potential clustering of patients between practices. | | | | | | |

eTable 11 presents risk differences per 100 patients treated and 95% confidence intervals for the relative association between prescription of varenicline compared to NRT and smoking cessation, stratified by mental disorder. Varenicline was associated with smoking cessation relative to NRT at all follow-ups in smokers with most mental disorders. Except for there was some evidence that varenicline was no more effective than NRT in smokers with bipolar at 3-months, and 1 to 4-years follow-up, and in smokers with schizophrenia at 4-years.

## eTable 12. Stratified by mental disorder: Instrumental variable regression risk difference per 100 patients and 95% confidence intervals for the association between prescription of varenicline versus NRT and smoking cessation at 3, 6 and 9-months, and 1, 2, and 4-years follow-up

| **Instrumental variable regression‡ models**  **Risk difference per 100 patients (95% confidence interval)** | | | | | | |
| --- | --- | --- | --- | --- | --- | --- |
|  | **3-months** | **6-months** | **9-months** | **1-year** | **2-years** | **4-years** |
| No mental disorder (N=136,654) | 3.63 (1.72 to 5.54) | 6.18 (4.18 to 8.18) | 6.56 (4.57 to 8.56) | 6.51 (4.54 to 8.48) | 5.35 (3.50 to 7.19) | 4.75 (2.97 to 6.53) |
| Any mental disorder (N=68,578) | 5.48 (3.11 to 7.85) | 7.25 (4.75 to 9.76) | 7.26 (4.74 to 9.77) | 6.81 (4.30 to 9.32) | 4.91 (2.42 to 7.40) | 3.59 (1.06 to 6.13) |
| Bipolar (N=1,735) | 4.32 (-15.64 to 24.28) | 17.75 (-4.70 to 40.20) | 10.92 (-12.09 to 33.93) | 12.90 (-10.69 to 36.48) | 10.84 (-13.64 to 35.32) | 10.02 (-15.77 to 35.81) |
| Depression (N=14,695) | 7.72 (3.41 to 12.03) | 9.52 (4.80 to 14.25) | 9.30 (4.61 to 13.99) | 8.60 (3.78 to 13.43) | 6.54 (1.62 to 11.46) | 5.90 (0.95 to 10.86) |
| Neurotic disorder (N=7,269) | 6.94 (1.43 to 12.44) | 6.14 (0.10 to 12.18) | 7.34 (0.99 to 13.70) | 6.57 (0.09 to 13.04) | 2.09 (-4.55 to 8.73) | 5.14 (-1.88 to 12.16) |
| Schizophrenia (N=4,125) | 4.04 (-7.91 to 15.99) | 3.40 (-9.62 to 16.43) | -2.02 (-15.65 to 11.61) | -4.84 (-18.73 to 9.05) | 11.17 (-3.35 to 25.70) | 8.53 (-7.15 to 24.21) |
| Anti-depressants (N=49,763) | 4.83 (2.23 to 7.42) | 6.40 (3.62 to 9.18) | 6.45 (3.63 to 9.26) | 6.25 (3.45 to 9.06) | 5.10 (2.18 to 8.01) | 3.43 (0.46 to 6.41) |
| Anti-psychotics (N=10,345) | 4.47 (-1.79 to 10.73) | 6.22 (-0.36 to 12.8) | 4.83 (-2.04 to 11.71) | 5.28 (-1.67 to 12.22) | 8.06 (0.67 to 15.46) | 4.26 (-3.32 to 11.84) |
| Hypnotics/anxiolytics (N=27,214) | 4.70 (1.36 to 8.04) | 6.91 (3.28 to 10.54) | 7.11 (3.38 to 10.85) | 6.54 (2.82 to 10.26) | 4.04 (0.21 to 7.86) | 5.08 (1.07 to 9.09) |
| Mood stabilisers (N=4,104) | 5.85 (-4.76 to 16.45) | 2.57 (-9.12 to 14.25) | 4.11 (-8.53 to 16.76) | 3.90 (-9.01 to 16.80) | 6.78 (-6.26 to 19.82) | 6.04 (-7.80 to 19.89) |
| ‡Partially adjusted estimates were adjusted for age, sex, year of prescription. Models were estimated using cluster robust standard errors to account for potential clustering of patients between practices. | | | | | | |

eTable 12 presents risk differences per 100 patients treated and 95% confidence intervals for the relative association between prescription of varenicline compared to NRT and smoking cessation, stratified by mental disorder, derived from instrumental variable models. The instrumental variable models showed evidence that varenicline was more effective than NRT in smokers with any mental disorder at all follow-ups, and in smokers prescribed antidepressants from 3-months to 2-years follow-up, this association attenuated by 4-years follow-up; however, estimates were imprecise, and this change was consistent with sampling variability. The instrumental variable models conducted in smokers with bipolar, depression, neurotic disorder, or schizophrenia, and in smokers prescribed antipsychotics, hypnotics/anxiolytics, or mood stabilisers were underpowered to treatment effect heterogeneity.

## eTable 13. Bland-Altman tests for a difference in multivariable instrumental variable regression coefficients for an association between varenicline and smoking cessation at 2-years follow-up: The difference between adjusted‡ risk differences and 95% intervals derived from patients with mental disorders, compared to smokers with no mental disorder*

|  | **P-value for difference in coefficients** |
| --- | --- |
| Any mental disorder (N=68,578) | 0.38 |
| Bipolar (N=1,735) | 0.36 |
| Depression (N=14,695) | 0.36 |
| Neurotic disorder (N=7,269) | 0.26 |
| Schizophrenia (N=4,125) | 0.29 |
| Anti-depressants (N=49,763) | 0.39 |
| Anti-psychotics (N=10,345) | 0.31 |
| Hypnotics/anxiolytics (N=27,214) | 0.33 |
| Mood stabilisers (N=4,104) | 0.39 |
| ‡Partially adjusted estimates were adjusted for age, sex, year of prescription. Models were estimated using cluster robust standard errors to account for potential clustering of patients between practices. **No mental disorder N= 136,654 | |

## eFigure 4. Bias plot: Standardised % bias across covariates before and after propensity score matching

eFigure 4 displays the percent bias difference between exposure groups, before and after propensity score matching. I.e. the percent difference of the sample means in the control and treatment groups, as a percentage of the square root of the average of the sample variances in each group.(1) The model achieved an excellent covariate balance between the treatment conditions.

## eFigure 5. Bias plot: Kernel density plots of propensity scores before and after matching

**Before matching**

**After matching**

Comparison of Kernel density estimation plots of groups’ propensity scores before and after matching indicates that there was an adequate common support region to conduct the propensity score matching procedure and that an excellent overlap of propensity score distributions was achieved after matching (eFigure 5).

## eFigure 6. Bias plot: Relative bias of linear regression and instrumental variable methods: binary and continuous outcomes

eFigure 6 shows the relative bias of linear regression and instrumental variable methods. Squares (■) indicate the bias component in the linear regression model, and triangles () indicate the bias component in the instrumental variable model.

# References

1. Leuven E, Sianesi B. PSMATCH2: STATA module to perform full Mahalanobis and propensity score matching, common support graphing, and covariate imbalance testing. Boston, MA, USA: Boston College Department of Economics; 2003.

2. Rosenbaum P, Rubin D. The central role of the propensity score in observational studies for causal effects. Biometrika. 1983;70(1):41–55.

3. Rosenbaum P, Rubin D. Reducing bias in observational studies using subclassification on the propensity score. J Am Stat Assoc. 1984;79(387):516–24.

4. Glynn RJ, Schneeweiss S, Stürmer T. Indications for propensity scores and review of their use in pharmacoepidemiology. Basic Clin Pharmacol Toxicol. 2006;98(3):253–9.

5. Taylor G, Girling A, McNeill A, Aveyard P. Does smoking cessation result in improved mental health? A comparison of regression modelling and propensity score matching. BMJ Open. 2015;

6. Taylor G, Taylor A, Munafò MR, McNeill A, Aveyard P. Does smoking reduction worsen mental health? A comparison of two observational approaches. BMJ Open. 2015;5(5):e007812.

7. Thomas KH, Martin RM, Davies NM, Metcalfe C, Windmeijer F, Gunnell D. Smoking cessation treatment and risk of depression, suicide, and self harm in the Clinical Practice Research Datalink: Prospective cohort study. BMJ. 2013;

8. Thoemmes F, Kim E. A systematic review of propensity score methods in the social sciences. Multivariate Behav Res. 2011;46(1):90–118.

9. King G, Zeng L. The dangers of extreme counterfactuals. Polit Anal. 2005;14(2):131–59.

10. Davies NM, Gunnell D, Thomas KH, Metcalfe C, Windmeijer F, Martin RM. Physicians’ prescribing preferences were a potential instrument for patients’ actual prescriptions of antidepressants. J Clin Epidemiol. 2013;

11. Clarke PS, Windmeijer F. Instrumental variable estimators for binary outcomes. Journal of the American Statistical Association. 2012.

12. Davies NM, Smith GD, Windmeijer F, Martin RM. COX-2 selective nonsteroidal anti-inflammatory drugs and risk of gastrointestinal tract complications and myocardial infarction: An instrumental variable analysis. Epidemiology. 2013;

13. Angrist JD, Imbens GW, Rubin DB. Identification of Causal Effects Using Instrumental Variables. J Am Stat Assoc. 1996;

14. Davies NM, Taylor GMJ, Taylor AE, Jones T, Martin RM, Munafò MR, et al. The effects of prescribing varenicline on two-year health outcomes: an observational cohort study using electronic medical records. Addiction. 2018;113(6).

15. Taylor GMJ, Taylor AE, Thomas KH, Jones T, Martin RM, Munafò MR, et al. The effectiveness of varenicline versus nicotine replacement therapy on long-term smoking cessation in primary care: A prospective cohort study of electronic medical records. Int J Epidemiol. 2017;46(6).

16. Hansen LP, Singleton KJ. Generalized instrumental variables estimation of nonlinear rational expectations models. Econom J Econom Soc. 1982;1269–86.

17. Clarke PS, Windmeijer F. Identification of causal effects on binary outcomes using structural mean models. Biostatistics. 2010;11(4):756–70.
