# Supplementary material for: Prescribing Prevalence, Effectiveness, and Mental Health Safety of Smoking Cessation Medicines in Patients With Mental Disorders
Source: Nicotine Tob Res. 2019 Jul 10;22(1):48–57. doi: 10.1093/ntr/ntz072 (PMC7073926; doi:10.1093/ntr/ntz072)
Supplement: ntz072_suppl_Supplementary-Material-2 [file ntz072_suppl_supplementary-material-2.docx]

**Supplementary material 2**

**The association between varenicline and mental health outcomes**

**List of tables**

[eTable 1. Number and percentage (%) of patients with an electronic medical record indicating depression at 3, 6 and 9-months, and 1, 2, and 4-years follow-up by exposure group, and by mental disorder* 3](#_Toc786049)

[eTable 2. Number and percentage (%) of patients with an electronic medical record indicating neurotic disorder at 3, 6 and 9-months, and 1, 2, and 4-years follow-up by exposure group, and by mental disorder* 4](#_Toc786050)

[eTable 3. Number and percentage (%) of patients with an electronic medical record indicating an antidepressant prescription at 3, 6 and 9-months, and 1, 2, and 4-years follow-up by exposure group, and by mental disorder 5](#_Toc786051)

[eTable 4. Number and percentage (%) of patients with an electronic medical record indicating a hypnotic/anxiolytic prescription at 3, 6 and 9-months, and 1, 2, and 4-years follow-up by exposure group, and by mental disorder 6](#_Toc786052)

[eTable 5. Stratified by mental disorder: Partially adjusted odds ratios and 95% confidence intervals for the association between prescription of varenicline versus NRT and depression at 3, 6 and 9-months, and 1, 2, and 4-years after prescription 7](#_Toc786053)

[eTable 6. Stratified by mental disorder: Partially adjusted odds ratios and 95% confidence intervals for the association between prescription of varenicline versus NRT and neurotic disorder at 3, 6 and 9-months, and 1, 2, and 4-years after prescription 8](#_Toc786054)

[eTable 7. Stratified by mental disorder: Partially adjusted odds ratios and 95% confidence intervals for the association between prescription of varenicline versus NRT and antidepressant prescription at 3, 6 and 9-months, and 1, 2, and 4-years after prescription 9](#_Toc786055)

[eTable 8. Stratified by mental disorder: Partially adjusted odds ratios and 95% confidence intervals for the association between prescription of varenicline versus NRT and hypnotic/anxiolytic prescription at 3, 6 and 9-months, and 1, 2, and 4-years after prescription 10](#_Toc786056)

[eTable 9. Stratified by mental disorder: Fully adjusted odds ratios and 95% confidence intervals for the association between prescription of varenicline versus NRT and depression at 3, 6 and 9-months, and 1, 2, and 4-years after prescription 11](#_Toc786057)

[eTable 10. Stratified by mental disorder: Fully adjusted odds ratios and 95% confidence intervals for the association between prescription of varenicline versus NRT and neurotic disorder at 3, 6 and 9-months, and 1, 2, and 4-years after prescription 12](#_Toc786058)

[eTable 11. Stratified by mental disorder: Fully adjusted odds ratios and 95% confidence intervals for the association between prescription of varenicline versus NRT and antidepressant prescription at 3, 6 and 9-months, and 1, 2, and 4-years after prescription 13](#_Toc786059)

[eTable 12. Stratified by mental disorder: Fully adjusted odds ratios and 95% confidence intervals for the association between prescription of varenicline versus NRT and hypnotic/anxiolytic prescription at 3, 6 and 9-months, and 1, 2, and 4-years after prescription 14](#_Toc786060)

[eTable 13. Stratified by mental disorder: Propensity score matched logistic regression odds ratios and 95% confidence intervals for the association between prescription of varenicline versus NRT and depression at 3, 6 and 9-months, and 1, 2, and 4-years follow-up 15](#_Toc786061)

[eTable 14. Stratified by mental disorder: Propensity score matched logistic regression odds ratios and 95% confidence intervals for the association between prescription of varenicline versus NRT and neurotic disorder at 3, 6 and 9-months, and 1, 2, and 4-years follow-up 16](#_Toc786062)

[eTable 15. Stratified by mental disorder: Propensity score matched logistic regression odds ratios and 95% confidence intervals for the association between prescription of varenicline versus NRT and antidepressant prescription at 3, 6 and 9-months, and 1, 2, and 4-years follow-up 17](#_Toc786063)

[eTable 16. Stratified by mental disorder: Propensity score matched logistic regression odds ratios and 95% confidence intervals for the association between prescription of varenicline versus NRT and hypnotic/anxiolytic prescription at 3, 6 and 9-months, and 1, 2, and 4-years follow-up 18](#_Toc786064)

[eTable 17. Stratified by mental disorder: Linear regression risk difference per 100 patients and 95% confidence intervals for the association between prescription of varenicline versus NRT and depression at 3, 6 and 9-months, and 1, 2, and 4-years follow-up 19](#_Toc786065)

[eTable 18. Stratified by mental disorder: Linear regression risk difference per 100 patients and 95% confidence intervals for the association between prescription of varenicline versus NRT and neurotic disorder at 3, 6 and 9-months, and 1, 2, and 4-years follow-up 20](#_Toc786066)

[eTable 19. Stratified by mental disorder: Linear regression risk difference per 100 patients and 95% confidence intervals for the association between prescription of varenicline versus NRT and antidepressant prescription at 3, 6 and 9-months, and 1, 2, and 4-years 21](#_Toc786067)

[eTable 20. Stratified by mental disorder: Linear regression risk difference per 100 patients and 95% confidence intervals for the association between prescription of varenicline versus NRT and hypnotic/anxiolytic prescription at 3, 6 and 9-months, and 1, 2, and 4-years 22](#_Toc786068)

[eTable 21. Stratified by mental disorder: Instrumental variable regression risk difference per 100 patients and 95% confidence intervals for the association between prescription of varenicline versus NRT and depression at 3, 6 and 9-months, and 1, 2, and 4-years follow-up 23](#_Toc786069)

[eTable 22. Stratified by mental disorder: Instrumental variable regression risk difference per 100 patients and 95% confidence intervals for the association between prescription of varenicline versus NRT and neurotic disorder at 3, 6 and 9-months, and 1, 2, and 4-years follow-up 24](#_Toc786070)

[eTable 23. Stratified by mental disorder: Instrumental variable regression risk difference per 100 patients and 95% confidence intervals for the association between prescription of varenicline versus NRT and antidepressant prescription at 3, 6 and 9-months, and 1, 2, and 4-years follow-up 25](#_Toc786071)

[eTable 24. Stratified by mental disorder: Instrumental variable regression risk difference per 100 patients and 95% confidence intervals for the association between prescription of varenicline versus NRT and hypnotic/anxiolytic prescription at 3, 6 and 9-months, and 1, 2, and 4-years follow-up 26](#_Toc786072)

## eTable 1. Number and percentage (%) of patients with an electronic medical record indicating depression at 3, 6 and 9-months, and 1, 2, and 4-years follow-up by exposure group, and by mental disorder*

|  | **Treatment** | **3-months** | **6-months** | **9-months** | **1-year** | **2-years** | **4-years** |
| --- | --- | --- | --- | --- | --- | --- | --- |
| No mental disorder | NRT (N=100,396) | 1,113 (1.1%) | 2,184 (2.2%) | 3,173 (3.2%) | 4,206 (4.2%) | 7,337 (7.3%) | 11,470 (11.4%) |
|  | Varenicline (N=56,461) | 445 (0.8%) | 1,063 (1.9%) | 1,591 (2.8%) | 2,102 (3.7%) | 3,711 (6.6%) | 5,873 (10.4%) |
| Any mental disorder | NRT (N=45,919) | 1,215 (2.6%) | 2,373 (5.2%) | 3,412 (7.4%) | 4,309 (9.4%) | 7,045 (15.3%) | 10,231 (22.3%) |
|  | Varenicline (N=15,370) | 370 (2.4%) | 752 (4.9%) | 1,087 (7.1%) | 1,400 (9.1%) | 2,372 (15.4%) | 3,439 (22.4%) |
| Bipolar | NRT (N=1,799) | 32 (2.1%) | 67 (4.1%) | 101 (6.1%) | 127 (8.1%) | 185 (12.1%) | 264 (17.1%) |
|  | Varenicline (N=213) | 6 (3.1%) | 10 (5.1%) | 11 (6.1%) | 13 (7.1%) | 25 (14.1%) | 36 (19.1%) |
| Neurotic disorder | NRT (N=6,453) | 151 (4.1%) | 279 (7.1%) | 395 (10.1%) | 465 (12.1%) | 717 (18.1%) | 1,003 (25.1%) |
|  | Varenicline (N=1,941) | 36 (3.1%) | 77 (6.1%) | 108 (8.1%) | 133 (10.1%) | 226 (18.1%) | 323 (25.1%) |
| Schizophrenia | NRT (N=4,263) | 62 (2.1%) | 122 (3.1%) | 175 (5.1%) | 216 (6.1%) | 373 (10.1%) | 557 (15.1%) |
|  | Varenicline (N=441) | 10 (3.1%) | 17 (4.1%) | 27 (7.1%) | 39 (10.1%) | 67 (17.1%) | 92 (24.1%) |
| Antidepressants | NRT (N=43,589) | 951 (3.1%) | 1,842 (6.1%) | 2,667 (8.1%) | 3,359 (11.1%) | 5,511 (17.1%) | 7,928 (25.1%) |
|  | Varenicline (N=13,167) | 274 (3.1%) | 561 (6.1%) | 798 (8.1%) | 1,033 (10.1%) | 1,756 (18.1%) | 2,503 (25.1%) |
| Antipsychotics | NRT (N=9,843) | 188 (2.1%) | 364 (4.1%) | 521 (6.1%) | 665 (8.1%) | 1,073 (13.1%) | 1,580 (19.1%) |
|  | Varenicline (N=1,986) | 33 (2.1%) | 79 (4.1%) | 118 (7.1%) | 146 (8.1%) | 241 (14.1%) | 348 (20.1%) |
| Hypnotics/anxiolytics | NRT (N=23,651) | 510 (3.1%) | 1,015 (5.1%) | 1,457 (8.1%) | 1,829 (10.1%) | 2,947 (15.1%) | 4,277 (22.1%) |
|  | Varenicline (N=7,640) | 163 (2.1%) | 332 (5.1%) | 476 (7.1%) | 600 (9.1%) | 974 (15.1%) | 1,430 (22.1%) |
| Mood stabilisers | NRT (N=4,079) | 63 (2.1%) | 116 (3.1%) | 169 (5.1%) | 221 (6.1%) | 378 (10.1%) | 583 (16.1%) |
|  | Varenicline (N=649) | 14 (2.1%) | 22 (4.1%) | 32 (6.1%) | 41 (7.1%) | 79 (14.1%) | 116 (20.1%) |

*Patients with a depression diagnosis within 365 days of baseline were excluded as change depression diagnoses are not regularly recorded in the CPRD.

eTable 1 shows that the rate of depression diagnoses was lower or the same at all follow-ups in patients prescribed varenicline compared to NRT for most mental disorders; except for in patients with any mental disorder at 2 and 4-years follow-up, in patients with schizophrenia or bipolar and in patients prescribed antipsychotics or mood stabilisers the varenicline group had higher rates of depression compared to NRT at all follow-ups, and there were higher rates of depression in patients prescribed varenicline at 2-years follow-up.

## eTable 2. Number and percentage (%) of patients with an electronic medical record indicating neurotic disorder at 3, 6 and 9-months, and 1, 2, and 4-years follow-up by exposure group, and by mental disorder*

|  | **Treatment** | **3-months** | **6-months** | **9-months** | **1-year** | **2-years** | **4-years** |
| --- | --- | --- | --- | --- | --- | --- | --- |
| No mental disorder | NRT (N=100,396) | 623 (0.6%) | 1,150 (1.1%) | 1,700 (1.7%) | 2,222 (2.2%) | 3,901 (3.9%) | 6,293 (6.3%) |
|  | Varenicline (N=56,461) | 250 (0.4%) | 553 (1.0%) | 790 (1.4%) | 1,052 (1.9%) | 1,896 (3.4%) | 3,120 (5.5%) |
| Any mental disorder | NRT (N=52,887) | 717 (1.4%) | 1,420 (2.7%) | 2,053 (3.9%) | 2,700 (5.1%) | 4,510 (8.5%) | 6,876 (13.0%) |
|  | Varenicline (N=17,176) | 193 (1.1%) | 412 (2.4%) | 618 (3.6%) | 803 (4.7%) | 1,366 (8.0%) | 2,057 (12.0%) |
| Bipolar | NRT (N=1,799) | 27 (2.1%) | 42 (3.1%) | 60 (4.1%) | 76 (5.1%) | 127 (8.1%) | 205 (12.1%) |
|  | Varenicline (N=213) | <5 (<0.5%) | <5 (<0.5%) | <5 (<0.5%) | <5 (<0.5%) | 8 (4.1%) | 14 (7.1%) |
| Depression | NRT (N=13,421) | 189 (2.1%) | 364 (3.1%) | 529 (5.1%) | 688 (6.1%) | 1,172 (11.1%) | 1,746 (16.1%) |
|  | Varenicline (N=3,747) | 35 (1.1%) | 80 (3.1%) | 126 (4.1%) | 172 (6.1%) | 295 (10.1%) | 442 (14.1%) |
| Schizophrenia | NRT (N=4,263) | 48 (1.1%) | 87 (2.1%) | 128 (3.1%) | 176 (4.1%) | 285 (7.1%) | 457 (11.1%) |
|  | Varenicline (N=441) | 7 (2.1%) | 15 (4.1%) | 16 (4.1%) | 21 (5.1%) | 38 (9.1%) | 49 (12.1%) |
| Antidepressants | NRT (N=43,589) | 558 (1.1%) | 1,116 (3.1%) | 1,618 (4.1%) | 2,130 (5.1%) | 3,588 (9.1%) | 5,428 (14.1%) |
|  | Varenicline (N=13,167) | 146 (1.1%) | 306 (3.1%) | 465 (4.1%) | 601 (5.1%) | 1,029 (9.1%) | 1,537 (13.1%) |
| Antipsychotics | NRT (N=9,843) | 135 (2.1%) | 254 (3.1%) | 367 (4.1%) | 475 (5.1%) | 778 (9.1%) | 1,197 (13.1%) |
|  | Varenicline (N=1,986) | 23 (1.1%) | 52 (3.1%) | 75 (4.1%) | 100 (5.1%) | 151 (8.1%) | 227 (12.1%) |
| Hypnotics/anxiolytics | NRT (N=23,651) | 343 (2.1%) | 688 (3.1%) | 977 (5.1%) | 1,268 (6.1%) | 2,057 (10.1%) | 3,083 (15.1%) |
|  | Varenicline (N=7,640) | 81 (1.1%) | 172 (3.1%) | 260 (4.1%) | 345 (5.1%) | 578 (8.1%) | 868 (13.1%) |
| Mood stabilisers | NRT (N=4,079) | 52 (1.1%) | 81 (2.1%) | 116 (3.1%) | 160 (4.1%) | 278 (7.1%) | 422 (11.1%) |
|  | Varenicline (N=649) | 9 (1.1%) | 13 (2.1%) | 17 (3.1%) | 17 (3.1%) | 31 (5.1%) | 57 (9.1%) |

*Patients with a neurotic disorder diagnosis within 365 days of baseline were excluded as change neurotic disorder diagnoses are not regularly recorded in the CPRD.

eTable 2 shows that the rate of neurotic disorder was lower or the same at all follow-ups in patients prescribed varenicline compared to NRT for all mental disorders, except for in patients with schizophrenia varenicline produced higher neurotic disorder rates.

## eTable 3. Number and percentage (%) of patients with an electronic medical record indicating an antidepressant prescription at 3, 6 and 9-months, and 1, 2, and 4-years follow-up by exposure group, and by mental disorder

|  | **Treatment** | **3-months** | **6-months** | **9-months** | **1-year** | **2-years** | **4-years** |
| --- | --- | --- | --- | --- | --- | --- | --- |
| No mental disorder | NRT (N=100,396) | 2,689 (2.7%) | 5,027 (5.0%) | 7,132 (7.1%) | 9,220 (9.2%) | 15,419 (15.4%) | 23,507 (23.4%) |
|  | Varenicline (N=56,461) | 1,061 (1.9%) | 2,324 (4.1%) | 3,496 (6.2%) | 4,580 (8.1%) | 8,065 (14.3%) | 12,472 (22.1%) |
| Any mental disorder | NRT (N=59,340) | 30,696 (51.7%) | 33,851 (57.0%) | 35,705 (60.2%) | 37,197 (62.7%) | 40,685 (68.6%) | 43,909 (74.0%) |
|  | Varenicline (N=19,117) | 7,687 (40.2%) | 8,818 (46.1%) | 9,561 (50.0%) | 10,140 (53.0%) | 11,650 (60.9%) | 13,001 (68.0%) |
| Bipolar | NRT (N=1,799) | 907 (50.4%) | 960 (53.4%) | 1,007 (56.0%) | 1,039 (57.8%) | 1,124 (62.5%) | 1,205 (67.0%) |
|  | Varenicline (N=213) | 92 (43.2%) | 103 (48.4%) | 108 (50.7%) | 112 (52.6%) | 126 (59.2%) | 139 (65.3%) |
| Depression | NRT (N=13,421) | 7,981 (59.5%) | 8,758 (65.3%) | 9,222 (68.7%) | 9,583 (71.4%) | 10,404 (77.5%) | 11,036 (82.2%) |
|  | Varenicline (N=3,747) | 1,697 (45.3%) | 1,944 (51.9%) | 2,081 (55.5%) | 2,209 (59.0%) | 2,494 (66.6%) | 2,751 (73.4%) |
| Neurotic disorder | NRT (N=6,453) | 3,291 (51.0%) | 3,645 (56.5%) | 3,900 (60.4%) | 4,064 (63.0%) | 4,464 (69.2%) | 4,812 (74.6%) |
|  | Varenicline (N=1,941) | 685 (35.3%) | 806 (41.5%) | 892 (46.0%) | 938 (48.3%) | 1,116 (57.5%) | 1,277 (65.8%) |
| Schizophrenia | NRT (N=4,263) | 2,006 (47.1%) | 2,120 (49.7%) | 2,199 (51.6%) | 2,261 (53.0%) | 2,441 (57.3%) | 2,616 (61.4%) |
|  | Varenicline (N=441) | 169 (38.3%) | 202 (45.8%) | 217 (49.2%) | 225 (51.0%) | 250 (56.7%) | 275 (62.4%) |
| Antidepressants | NRT (N=43,589) | 29,758 (68.3%) | 32,211 (73.9%) | 33,466 (76.8%) | 34,418 (79.0%) | 36,378 (83.5%) | 37,962 (87.1%) |
|  | Varenicline (N=13,167) | 7,414 (56.3%) | 8,246 (62.6%) | 8,734 (66.3%) | 9,093 (69.1%) | 9,973 (75.7%) | 10,664 (81.0%) |
| Antipsychotics | NRT (N=9,843) | 5,018 (51.0%) | 5,401 (54.9%) | 5,659 (57.5%) | 5,833 (59.3%) | 6,303 (64.0%) | 6,776 (68.8%) |
|  | Varenicline (N=1,986) | 744 (37.5%) | 865 (43.6%) | 941 (47.4%) | 991 (49.9%) | 1,131 (56.9%) | 1,264 (63.6%) |
| Hypnotics/anxiolytics | NRT (N=23,651) | 11,584 (49.0%) | 12,781 (54.0%) | 13,522 (57.2%) | 14,137 (59.8%) | 15,697 (66.4%) | 17,180 (72.6%) |
|  | Varenicline (N=7,640) | 2,632 (34.5%) | 3,079 (40.3%) | 3,366 (44.1%) | 3,620 (47.4%) | 4,281 (56.0%) | 4,905 (64.2%) |
| Mood stabilisers | NRT (N=4,079) | 1,862 (45.6%) | 1,998 (49.0%) | 2,073 (50.8%) | 2,140 (52.5%) | 2,339 (57.3%) | 2,548 (62.5%) |
|  | Varenicline (N=649) | 275 (42.4%) | 302 (46.5%) | 318 (49.0%) | 336 (51.8%) | 372 (57.3%) | 403 (62.1%) |

eTable 3 shows that the rate of antidepressant prescriptions was lower at all follow-ups in patients prescribed varenicline compared to NRT for all mental disorders, except for in patients with schizophrenia there was a higher rate of antidepressant prescriptions at 4-years in patients prescribed varenicline.

## eTable 4. Number and percentage (%) of patients with an electronic medical record indicating a hypnotic/anxiolytic prescription at 3, 6 and 9-months, and 1, 2, and 4-years follow-up by exposure group, and by mental disorder

|  | **Treatment** | **3-months** | **6-months** | **9-months** | **1-year** | **2-years** | **4-years** |
| --- | --- | --- | --- | --- | --- | --- | --- |
| No mental disorder | NRT (N=100,396) | 1,801 (1.8%) | 3,222 (3.2%) | 4,576 (4.6%) | 5,896 (5.9%) | 9,936 (9.9%) | 15,493 (15.4%) |
|  | Varenicline (N=56,461) | 749 (1.3%) | 1,548 (2.7%) | 2,305 (4.1%) | 3,006 (5.3%) | 5,256 (9.3%) | 8,259 (14.6%) |
| Any mental disorder | NRT (N=59,340) | 12,647 (21.3%) | 15,410 (26.0%) | 17,377 (29.3%) | 19,052 (32.1%) | 23,228 (39.1%) | 27,638 (46.6%) |
|  | Varenicline (N=19,117) | 3,142 (16.4%) | 4,037 (21.1%) | 4,753 (24.9%) | 5,319 (27.8%) | 6,749 (35.3%) | 8,187 (42.8%) |
| Bipolar | NRT (N=1,799) | 602 (33.5%) | 685 (38.1%) | 749 (41.6%) | 806 (44.8%) | 925 (51.4%) | 1,045 (58.1%) |
|  | Varenicline (N=213) | 54 (25.4%) | 63 (29.6%) | 71 (33.3%) | 78 (36.6%) | 96 (45.1%) | 118 (55.4%) |
| Depression | NRT (N=13,421) | 2,239 (16.7%) | 2,885 (21.5%) | 3,339 (24.9%) | 3,745 (27.9%) | 4,761 (35.5%) | 5,858 (43.6%) |
|  | Varenicline (N=3,747) | 468 (12.5%) | 627 (16.7%) | 787 (21.0%) | 882 (23.5%) | 1,172 (31.3%) | 1,463 (39.0%) |
| Neurotic disorder | NRT (N=6,453) | 1,385 (21.5%) | 1,738 (26.9%) | 1,982 (30.7%) | 2,193 (34.0%) | 2,701 (41.9%) | 3,180 (49.3%) |
|  | Varenicline (N=1,941) | 300 (15.5%) | 407 (21.0%) | 495 (25.5%) | 552 (28.4%) | 699 (36.0%) | 856 (44.1%) |
| Schizophrenia | NRT (N=4,263) | 1,216 (28.5%) | 1,405 (33.0%) | 1,528 (35.8%) | 1,646 (38.6%) | 1,905 (44.7%) | 2,173 (51.0%) |
|  | Varenicline (N=441) | 99 (22.4%) | 119 (27.0%) | 128 (29.0%) | 144 (32.7%) | 175 (39.7%) | 204 (46.3%) |
| Antidepressants | NRT (N=43,589) | 8,860 (20.3%) | 10,819 (24.8%) | 12,257 (28.1%) | 13,492 (31.0%) | 16,609 (38.1%) | 19,995 (45.9%) |
|  | Varenicline (N=13,167) | 1,981 (15.0%) | 2,562 (19.5%) | 3,044 (23.1%) | 3,424 (26.0%) | 4,457 (33.8%) | 5,479 (41.6%) |
| Antipsychotics | NRT (N=9,843) | 2,923 (29.7%) | 3,371 (34.2%) | 3,699 (37.6%) | 3,972 (40.4%) | 4,631 (47.0%) | 5,262 (53.5%) |
|  | Varenicline (N=1,986) | 379 (19.1%) | 468 (23.6%) | 534 (26.9%) | 596 (30.0%) | 728 (36.7%) | 872 (43.9%) |
| Hypnotics/anxiolytics | NRT (N=23,651) | 11,296 (47.8%) | 12,915 (54.6%) | 13,873 (58.7%) | 14,614 (61.8%) | 16,218 (68.6%) | 17,489 (73.9%) |
|  | Varenicline (N=7,640) | 2,803 (36.7%) | 3,355 (43.9%) | 3,734 (48.9%) | 4,017 (52.6%) | 4,571 (59.8%) | 5,045 (66.0%) |
| Mood stabilisers | NRT (N=4,079) | 1,159 (28.4%) | 1,336 (32.8%) | 1,464 (35.9%) | 1,557 (38.2%) | 1,799 (44.1%) | 2,063 (50.6%) |
|  | Varenicline (N=649) | 143 (22.0%) | 172 (26.5%) | 194 (29.9%) | 203 (31.3%) | 245 (37.8%) | 292 (45.0%) |

eTable 4 shows that the rate of hypnotic/anxiolytic prescriptions was lower at all follow-ups in patients prescribed varenicline compared to NRT for all mental disorders.

## eTable 5. Stratified by mental disorder: Partially adjusted odds ratios and 95% confidence intervals for the association between prescription of varenicline versus NRT and depression at 3, 6 and 9-months, and 1, 2, and 4-years after prescription

| **Logistic regression**  **Partially adjusted odds ratio (95% confidence interval) ‡*** | | | | | | |
| --- | --- | --- | --- | --- | --- | --- |
|  | **3-months** | **6-months** | **9-months** | **1-year** | **2-years** | **4-years** |
| No mental disorder (N=156,857) | 0.71 (0.63 to 0.8) | 0.87 (0.8 to 0.94) | 0.89 (0.84 to 0.95) | 0.89 (0.84 to 0.94) | 0.91 (0.87 to 0.95) | 0.96 (0.93 to 1) |
| Any mental disorder (N= 61,289) | 0.89 (0.79 to 1) | 0.93 (0.85 to 1.01) | 0.93 (0.86 to 1) | 0.95 (0.89 to 1.02) | 1 (0.94 to 1.06) | 1.03 (0.98 to 1.08) |
| Bipolar (N=1,766) | 1.84 (0.77 to 4.44) | 1.49 (0.75 to 2.96) | 1 (0.53 to 1.9) | 0.94 (0.52 to 1.7) | 1.27 (0.79 to 2.03) | 1.29 (0.85 to 1.95) |
| Neurotic disorder (N=5,243) | 0.72 (0.49 to 1.07) | 0.84 (0.64 to 1.1) | 0.83 (0.66 to 1.04) | 0.87 (0.7 to 1.06) | 0.96 (0.81 to 1.14) | 1 (0.86 to 1.16) |
| Schizophrenia (N=4,156) | 1.45 (0.74 to 2.86) | 1.27 (0.76 to 2.13) | 1.45 (0.93 to 2.24) | 1.73 (1.2 to 2.48) | 1.84 (1.38 to 2.47) | 1.78 (1.39 to 2.29) |
| Anti-depressants (N=41,515) | 0.9 (0.78 to 1.03) | 0.95 (0.86 to 1.05) | 0.93 (0.85 to 1.01) | 0.96 (0.88 to 1.04) | 1.01 (0.95 to 1.08) | 1.03 (0.97 to 1.09) |
| Anti-psychotics (N=10,198) | 0.83 (0.57 to 1.2) | 1.05 (0.81 to 1.35) | 1.09 (0.88 to 1.34) | 1.04 (0.86 to 1.26) | 1.08 (0.92 to 1.26) | 1.08 (0.95 to 1.24) |
| Hypnotics/anxiolytics (N=25,764) | 0.91 (0.76 to 1.1) | 0.94 (0.82 to 1.07) | 0.94 (0.84 to 1.05) | 0.94 (0.86 to 1.04) | 0.96 (0.88 to 1.04) | 0.99 (0.92 to 1.07) |
| Mood stabilisers (N=4,223) | 1.46 (0.81 to 2.62) | 1.28 (0.81 to 2.02) | 1.26 (0.86 to 1.84) | 1.23 (0.88 to 1.72) | 1.42 (1.1 to 1.85) | 1.41 (1.11 to 1.79) |
| ‡Partially adjusted models were adjusted for: age, sex, year of first prescription. Models were estimated using cluster robust standard errors to account for potential clustering of patients between practices. *Patients with a depression diagnosis within 365 days of baseline were excluded as change depression diagnoses are not regularly recorded in the CPRD. | | | | | | |

eTable 5 presents partially adjusted odds ratios and 95% confidence intervals for the relative association between prescription of varenicline compared to NRT and depression diagnosis, stratified by mental disorder. In patients with no mental disorder varenicline was associated with decreased odds of depression at 3, 6, 9-months, and 1, 2, and 4-years follow-up. There was no association between varenicline and depression in patients with any mental disorder bipolar disorder or neurotic disorder at any follow-up, patients with schizophrenia at 3, 9 and 9-months follow-up, and in patients prescribed anti-depressants, anti-psychotics or hypnotics/anxiolytics at 3, 6, 9-months, and 1, 2, and 4-years follow-up, and in patients prescribed mood stabilisers at 3, 6 and 9-months and 1-year follow-up. Varenicline was associated with increased odds of depression in patients with schizophrenia at 1, 2 and 4 years, in patients prescribed mood stabilisers at 2 and 4-years follow-up.

## eTable 6. Stratified by mental disorder: Partially adjusted odds ratios and 95% confidence intervals for the association between prescription of varenicline versus NRT and neurotic disorder at 3, 6 and 9-months, and 1, 2, and 4-years after prescription

| **Logistic regression**  **Partially adjusted odds ratio (95% confidence interval) ‡*** | | | | | | |
| --- | --- | --- | --- | --- | --- | --- |
|  | **3-months** | **6-months** | **9-months** | **1-year** | **2-years** | **4-years** |
| No mental disorder (N=156,857) | 0.72 (0.61 to 0.84) | 0.85 (0.76 to 0.95) | 0.82 (0.75 to 0.9) | 0.85 (0.78 to 0.92) | 0.88 (0.82 to 0.93) | 0.93 (0.88 to 0.98) |
| Any mental disorder (N=70,063) | 0.82 (0.69 to 0.96) | 0.9 (0.8 to 1) | 0.93 (0.84 to 1.02) | 0.91 (0.84 to 0.99) | 0.93 (0.87 to 1) | 0.94 (0.89 to 1) |
| Bipolar (N=1,914) | 0.36 (0.08 to 1.55) | 0.19 (0.03 to 1.37) | 0.27 (0.07 to 1.12) | 0.43 (0.16 to 1.19) | 0.5 (0.24 to 1.05) | 0.57 (0.32 to 1) |
| Depression (N=14,017) | 0.68 (0.46 to 0.98) | 0.81 (0.62 to 1.05) | 0.87 (0.7 to 1.07) | 0.9 (0.76 to 1.08) | 0.91 (0.79 to 1.05) | 0.94 (0.83 to 1.06) |
| Schizophrenia (N=4,387) | 1.43 (0.63 to 3.25) | 1.71 (0.94 to 3.1) | 1.23 (0.7 to 2.16) | 1.16 (0.71 to 1.88) | 1.32 (0.92 to 1.89) | 1.07 (0.78 to 1.46) |
| Anti-depressants (N=50,853) | 0.85 (0.71 to 1.02) | 0.9 (0.79 to 1.03) | 0.95 (0.85 to 1.05) | 0.92 (0.84 to 1.01) | 0.95 (0.88 to 1.02) | 0.95 (0.89 to 1.02) |
| Anti-psychotics (N=10,803) | 0.78 (0.5 to 1.21) | 1 (0.73 to 1.36) | 0.98 (0.76 to 1.28) | 1.01 (0.81 to 1.27) | 0.94 (0.77 to 1.13) | 0.93 (0.79 to 1.09) |
| Hypnotics/anxiolytics (N=27,491) | 0.69 (0.54 to 0.89) | 0.74 (0.62 to 0.88) | 0.78 (0.68 to 0.91) | 0.8 (0.7 to 0.9) | 0.83 (0.75 to 0.91) | 0.84 (0.77 to 0.92) |
| Mood stabilisers (N=4,469) | 1.05 (0.52 to 2.15) | 0.98 (0.54 to 1.78) | 0.9 (0.54 to 1.52) | 0.65 (0.39 to 1.08) | 0.67 (0.45 to 0.99) | 0.84 (0.62 to 1.14) |
| ‡ Partially adjusted models were adjusted for: age, sex, year of first prescription. Models were estimated using cluster robust standard errors to account for potential clustering of patients between practices. *Patients with a neurotic disorder diagnosis within 365 days of baseline were excluded as change neurotic disorder diagnoses are not regularly recorded in the CPRD. | | | | | | |

eTable 6 presents partially adjusted odds ratios and 95% confidence intervals for the relative association between prescription of varenicline compared to NRT and neurotic disorder diagnosis, stratified by mental disorder. There was no evidence for an association between varenicline and increased odds of neurotic disorder. Varenicline was associated with decreased odds of neurotic disorder for in patients with no mental disorder at 3, 6, and 9-months and 1, 2 and 4-years follow-up, any mental disorder at 3, and 6-months and 1, 2 and 4-years follow-up, in patients with bipolar at 4-years follow-up, in patients with depression at 3-months follow-up, in patients prescribed hypnotics/anxiolytics at 3, 6, and 9-months and 1, 2 and 4-years follow-up, and in patients prescribed mood stabilisers at 2-years follow-up. There was associated with lower odds of neurotic disorder in all other patient groups.

## eTable 7. Stratified by mental disorder: Partially adjusted odds ratios and 95% confidence intervals for the association between prescription of varenicline versus NRT and antidepressant prescription at 3, 6 and 9-months, and 1, 2, and 4-years after prescription

| **Logistic regression**  **Partially adjusted odds ratio (95% confidence interval) ‡** | | | | | | |
| --- | --- | --- | --- | --- | --- | --- |
|  | **3-months** | **6-months** | **9-months** | **1-year** | **2-years** | **4-years** |
| No mental disorder (N=156,857) | 0.67 (0.62 to 0.72) | 0.79 (0.75 to 0.83) | 0.84 (0.8 to 0.88) | 0.85 (0.82 to 0.88) | 0.92 (0.89 to 0.95) | 0.98 (0.95 to 1) |
| Any mental disorder (N=78,457) | 0.6 (0.58 to 0.62) | 0.62 (0.6 to 0.64) | 0.64 (0.61 to 0.66) | 0.64 (0.62 to 0.67) | 0.69 (0.67 to 0.72) | 0.74 (0.71 to 0.76) |
| Bipolar (N=2,012) | 0.73 (0.55 to 0.97) | 0.81 (0.61 to 1.07) | 0.79 (0.6 to 1.06) | 0.79 (0.59 to 1.06) | 0.85 (0.63 to 1.14) | 0.91 (0.67 to 1.24) |
| Depression (N=17,168) | 0.52 (0.49 to 0.57) | 0.53 (0.49 to 0.58) | 0.53 (0.49 to 0.57) | 0.54 (0.49 to 0.58) | 0.55 (0.5 to 0.59) | 0.58 (0.53 to 0.63) |
| Neurotic disorder (N=8,394) | 0.5 (0.45 to 0.56) | 0.53 (0.47 to 0.58) | 0.54 (0.48 to 0.59) | 0.53 (0.47 to 0.58) | 0.58 (0.52 to 0.65) | 0.65 (0.58 to 0.72) |
| Schizophrenia (N=4,704) | 0.67 (0.55 to 0.82) | 0.83 (0.68 to 1.01) | 0.88 (0.72 to 1.07) | 0.89 (0.73 to 1.09) | 0.94 (0.77 to 1.15) | 1.02 (0.83 to 1.25) |
| Anti-depressants (N=56,756) | 0.58 (0.55 to 0.6) | 0.57 (0.54 to 0.59) | 0.57 (0.55 to 0.6) | 0.57 (0.55 to 0.6) | 0.6 (0.57 to 0.63) | 0.63 (0.6 to 0.67) |
| Anti-psychotics (N=11,829) | 0.55 (0.5 to 0.61) | 0.61 (0.55 to 0.67) | 0.64 (0.58 to 0.71) | 0.66 (0.59 to 0.72) | 0.71 (0.64 to 0.79) | 0.77 (0.69 to 0.85) |
| Hypnotics/anxiolytics (N=31,291) | 0.52 (0.49 to 0.55) | 0.55 (0.52 to 0.58) | 0.56 (0.53 to 0.6) | 0.58 (0.55 to 0.61) | 0.62 (0.58 to 0.65) | 0.66 (0.62 to 0.7) |
| Mood stabilisers (N=4,728) | 0.83 (0.7 to 0.99) | 0.87 (0.73 to 1.03) | 0.89 (0.75 to 1.06) | 0.93 (0.78 to 1.11) | 0.96 (0.81 to 1.15) | 0.96 (0.81 to 1.15) |
| ‡ Partially adjusted models were adjusted for: age, sex, year of first prescription. Models were estimated using cluster robust standard errors to account for potential clustering of patients between practices. | | | | | | |

eTable 7 presents partially adjusted odds ratios and 95% confidence intervals for the relative association between prescription of varenicline compared to NRT and antidepressant prescription, stratified by mental disorder. There was no association between varenicline and antidepressant prescription in patients with bipolar and schizophrenia at 6 and 9-months, and 1, 2 and 4-years follow-up, and in patients prescribed mood stabilisers at all follow-ups. Varenicline was associated with lower odds of antidepressant prescription for remaining groups/follow-ups. There was no evidence for an association between varenicline and increased odds of antidepressant prescription.

## eTable 8. Stratified by mental disorder: Partially adjusted odds ratios and 95% confidence intervals for the association between prescription of varenicline versus NRT and hypnotic/anxiolytic prescription at 3, 6 and 9-months, and 1, 2, and 4-years after prescription

| **Logistic regression**  **Partially adjusted odds ratio (95% confidence interval) †** | | | | | | |
| --- | --- | --- | --- | --- | --- | --- |
|  | **3-months** | **6-months** | **9-months** | **1-year** | **2-years** | **4-years** |
| No mental disorder (N=156,857) | 0.75 (0.69 to 0.82) | 0.87 (0.81 to 0.92) | 0.91 (0.86 to 0.96) | 0.93 (0.88 to 0.97) | 0.98 (0.94 to 1.02) | 1.03 (1 to 1.06) |
| Any mental disorder (N=78,457) | 0.76 (0.72 to 0.79) | 0.79 (0.76 to 0.82) | 0.82 (0.79 to 0.86) | 0.84 (0.81 to 0.87) | 0.88 (0.85 to 0.91) | 0.9 (0.87 to 0.94) |
| Bipolar (N=2,012) | 0.66 (0.48 to 0.92) | 0.67 (0.49 to 0.91) | 0.69 (0.51 to 0.93) | 0.7 (0.52 to 0.94) | 0.76 (0.56 to 1.01) | 0.9 (0.68 to 1.21) |
| Depression (N=17,168) | 0.72 (0.64 to 0.8) | 0.73 (0.67 to 0.81) | 0.8 (0.73 to 0.88) | 0.8 (0.73 to 0.87) | 0.84 (0.78 to 0.91) | 0.86 (0.8 to 0.92) |
| Neurotic disorder (N=8,394) | 0.68 (0.59 to 0.79) | 0.73 (0.64 to 0.83) | 0.78 (0.7 to 0.88) | 0.79 (0.7 to 0.88) | 0.8 (0.72 to 0.89) | 0.84 (0.76 to 0.94) |
| Schizophrenia (N=4,704) | 0.71 (0.56 to 0.9) | 0.73 (0.59 to 0.92) | 0.72 (0.57 to 0.89) | 0.75 (0.61 to 0.93) | 0.8 (0.65 to 0.97) | 0.82 (0.67 to 1) |
| Anti-depressants (N=56,756) | 0.71 (0.67 to 0.75) | 0.74 (0.7 to 0.78) | 0.78 (0.74 to 0.82) | 0.79 (0.76 to 0.83) | 0.85 (0.81 to 0.89) | 0.87 (0.84 to 0.91) |
| Anti-psychotics (N=11,829) | 0.55 (0.49 to 0.62) | 0.58 (0.52 to 0.65) | 0.6 (0.54 to 0.67) | 0.62 (0.56 to 0.69) | 0.64 (0.58 to 0.71) | 0.68 (0.62 to 0.75) |
| Hypnotics/anxiolytics (N=31,291) | 0.66 (0.62 to 0.7) | 0.68 (0.64 to 0.71) | 0.7 (0.66 to 0.74) | 0.71 (0.67 to 0.75) | 0.71 (0.67 to 0.75) | 0.72 (0.68 to 0.77) |
| Mood stabilisers (N=4,728) | 0.69 (0.56 to 0.85) | 0.72 (0.6 to 0.88) | 0.75 (0.62 to 0.9) | 0.72 (0.6 to 0.87) | 0.76 (0.64 to 0.9) | 0.8 (0.68 to 0.95) |
| ‡ Partially adjusted models were adjusted for: age, sex, year of first prescription. Models were estimated using cluster robust standard errors to account for potential clustering of patients between practices. | | | | | | |

eTable 8 presents partially adjusted odds ratios and 95% confidence intervals for the relative association between prescription of varenicline compared to NRT and hypnotic/anxiolytic prescription, stratified by mental disorder. There was no association in patients with no mental disorder and bipolar at 2 and 4-years follow-up. Varenicline was associated with lower odds of hypnotic/anxiolytic prescription for remaining groups/follow-ups. There was no evidence for an association between varenicline and increased odds of hypnotic/anxiolytic prescription.

## eTable 9. Stratified by mental disorder: Fully adjusted odds ratios and 95% confidence intervals for the association between prescription of varenicline versus NRT and depression at 3, 6 and 9-months, and 1, 2, and 4-years after prescription

| **Logistic regression**  **Fully adjusted odds ratio (95% confidence interval) ‡*** | | | | | | |
| --- | --- | --- | --- | --- | --- | --- |
|  | **3-months** | **6-months** | **9-months** | **1-year** | **2-years** | **4-years** |
| No mental disorder (N=156,857) | 0.73 (0.65 to 0.82) | 0.90 (0.83 to 0.97) | 0.92 (0.86 to 0.98) | 0.91 (0.86 to 0.97) | 0.93 (0.89 to 0.98) | 0.99 (0.95 to 1.02) |
| Any mental disorder (N=61,289) | 0.91 (0.81 to 1.03) | 0.96 (0.88 to 1.05) | 0.97 (0.90 to 1.04) | 0.98 (0.92 to 1.05) | 1.03 (0.97 to 1.09) | 1.06 (1.00 to 1.11) |
| Bipolar (N=1,766) | 1.47 (0.56 to 3.84) | 1.40 (0.69 to 2.85) | 0.92 (0.48 to 1.80) | 0.88 (0.47 to 1.63) | 1.18 (0.72 to 1.94) | 1.22 (0.79 to 1.89) |
| Neurotic disorder (N=5,243) | 0.78 (0.52 to 1.17) | 0.93 (0.70 to 1.23) | 0.91 (0.73 to 1.15) | 0.96 (0.78 to 1.18) | 1.06 (0.89 to 1.25) | 1.08 (0.93 to 1.26) |
| Schizophrenia (N=4,156) | 1.28 (0.65 to 2.53) | 1.19 (0.74 to 1.99) | 1.35 (0.87 to 2.08) | 1.64 (1.14 to 2.37) | 1.68 (1.24 to 2.29) | 1.65 (1.27 to 2.15) |
| Anti-depressants (N=41,515) | 0.92 (0.80 to 1.06) | 0.99 (0.90 to 1.09) | 0.97 (0.88 to 1.06) | 0.99 (0.91 to 1.08) | 1.04 (0.97 to 1.11) | 1.06 (1.00 to 1.13) |
| Anti-psychotics (N=10,128) | 0.80 (0.55 to 1.17) | 1.05 (0.81 to 1.37) | 1.11 (0.89 to 1.38) | 1.05 (0.86 to 1.28) | 1.09 (0.93 to 1.29) | 1.09 (0.95 to 1.26) |
| Hypnotics/anxiolytics (N=25,764) | 0.96 (0.79 to 1.17) | 1.00 (0.88 to 1.15) | 1.01 (0.91 to 1.13) | 1.01 (0.91 to 1.11) | 1.02 (0.94 to 1.11) | 1.06 (0.98 to 1.14) |
| Mood stabilisers (N=4,223) | 1.41 (0.99 to 2.52) | 1.22 (0.78 to 1.93) | 1.25 (0.86 to 1.84) | 1.21 (0.86 to 1.70) | 1.41 (1.07 to 1.86) | 1.37 (1.07 to 1.76) |
| ‡Fully adjusted models were adjusted for: all baseline covariates. Models were estimated using cluster robust standard errors to account for potential clustering of patients between practices. *Patients with a depression diagnosis within 365 days of baseline were excluded as change in depression diagnoses are not regularly recorded in the CPRD. | | | | | | |

eTable 9 presents fully adjusted odds ratios and 95% confidence intervals for the relative association between prescription of varenicline compared to NRT and depression diagnosis, stratified by mental disorder. Varenicline was associated with lower odds of depression in patients with no mental disorder at 3, 6 and 9-months and 1 and 2-years.

Varenicline was associated with increased odds of depression in patients with any mental disorder at 4-years, in patients with schizophrenia at 1, 2 and 4-years follow-up, in patients prescribed anti-depressants at 4-years follow-up and in patients prescribed mood stabilisers at 2 and 4-years. There was no association between varenicline and depression at follow-up in all other groups/follow-ups.

## eTable 10. Stratified by mental disorder: Fully adjusted odds ratios and 95% confidence intervals for the association between prescription of varenicline versus NRT and neurotic disorder at 3, 6 and 9-months, and 1, 2, and 4-years after prescription

| **Logistic regression**  **Fully adjusted odds ratio (95% confidence interval) ‡*** | | | | | | |
| --- | --- | --- | --- | --- | --- | --- |
|  | **3-months** | **6-months** | **9-months** | **1-year** | **2-years** | **4-years** |
| No mental disorder (N=156,857) | 0.74 (0.64 to 0.86) | 0.87 (0.78 to 0.97) | 0.85 (0.77 to 0.93) | 0.87 (0.80 to 0.94) | 0.90 (0.85 to 0.96) | 0.95 (0.90 to 1.00) |
| Any mental disorder (N=70,063) | 0.88 (0.75 to 1.03) | 0.95 (0.85 to 1.06) | 0.98 (0.89 to 1.08) | 0.96 (0.89 to 1.05) | 0.98 (0.92 to 1.05) | 0.99 (0.93 to 1.05) |
| Bipolar (N=2,004) | 0.36 (0.08 to 1.62) | 0.47 (0.16 to 1.36) | 0.46 (0.18 to 1.16) | 0.57 (0.28 to 1.19) | 0.67 (0.39 to 1.15) | 0.71 (0.46 to 1.11) |
| Depression (N=14,017) | 0.73 (0.50 to 1.06) | 0.87 (0.67 to 1.13) | 0.92 (0.75 to 1.14) | 0.96 (0.80 to 1.14) | 0.96 (0.84 to 1.11) | 0.98 (0.87 to 1.10) |
| Schizophrenia (N=4,387) | 1.49 (0.65 to 3.44) | 1.66 (0.89 to 3.09) | 1.19 (0.66 to 2.12) | 1.12 (0.68 to 1.86) | 1.27 (0.88 to 1.84) | 1.02 (0.73 to 1.42) |
| Anti-depressants (N=50,853) | 0.92 (0.77 to 1.10) | 0.96 (0.84 to 1.10) | 1.00 (0.90 to 1.12) | 0.98 (0.89 to 1.08) | 1.00 (0.92 to 1.08) | 1.00 (0.94 to 1.07) |
| Anti-psychotics (N=10,803) | 0.87 (0.56 to 1.36) | 1.06 (0.77 to 1.46) | 1.04 (0.79 to 1.36) | 1.07 (0.85 to 1.36) | 0.99 (0.81 to 1.20) | 0.98 (0.83 to 1.16) |
| Hypnotics/anxiolytics (N=27,491) | 0.78 (0.61 to 1.00) | 0.81 (0.68 to 0.97) | 0.87 (0.75 to 1.02) | 0.88 (0.78 to 1.01) | 0.91 (0.82 to 1.01) | 0.92 (0.84 to 1.01) |
| Mood stabilisers (N=4,469) | 1.19 (0.58 to 2.44) | 1.08 (0.58 to 1.98) | 0.98 (0.58 to 1.67) | 0.72 (0.43 to 1.20) | 0.71 (0.48 to 1.05) | 0.91 (0.67 to 1.23) |
| ‡ Fully adjusted models were adjusted for: all baseline covariates. Models were estimated using cluster robust standard errors to account for potential clustering of patients between practices. *Patients with a neurotic disorder diagnosis within 365 days of baseline were excluded as change neurotic disorder diagnoses are not regularly recorded in the CPRD. | | | | | | |

eTable 10 presents fully adjusted odds ratios and 95% confidence intervals for the relative association between prescription of varenicline compared to NRT and neurotic disorder, stratified by mental disorder. There was no association between varenicline and neurotic disorder at most follow-ups. Varenicline was associated with lower odds of neurotic disorder in patients with no mental disorder at 3, 6 and 9-months, at 1 and 2-years, and in patients prescribed hypnotics/anxiolytics at 6-months. There was no evidence for an association between varenicline and increased odds of neurotic disorder.

## eTable 11. Stratified by mental disorder: Fully adjusted odds ratios and 95% confidence intervals for the association between prescription of varenicline versus NRT and antidepressant prescription at 3, 6 and 9-months, and 1, 2, and 4-years after prescription

| **Logistic regression**  **Fully adjusted odds ratio (95% confidence interval) ‡** | | | | | | |
| --- | --- | --- | --- | --- | --- | --- |
|  | **3-months** | **6-months** | **9-months** | **1-year** | **2-years** | **4-years** |
| No mental disorder (N=156,857) | 0.70 (0.65 to 0.75) | 0.82 (0.78 to 0.86) | 0.87 (0.83 to 0.91) | 0.88 (0.85 to 0.92) | 0.95 (0.92 to 0.98) | 1.01 (0.99 to 1.04) |
| Any mental disorder (N=78,457) | 0.63 (0.60 to 0.65) | 0.64 (0.62 to 0.67) | 0.66 (0.64 to 0.69) | 0.67 (0.65 to 0.70) | 0.73 (0.70 to 0.76) | 0.79 (0.76 to 0.82) |
| Bipolar (N=2,004) | 0.74 (0.52 to 1.05) | 0.86 (0.60 to 1.22) | 0.85 (0.59 to 1.22) | 0.83 (0.58 to 1.20) | 0.87 (0.60 to 1.26) | 0.95 (0.64 to 1.42) |
| Depression (N=17,168) | 0.55 (0.51 to 0.60) | 0.56 (0.51 to 0.61) | 0.55 (0.51 to 0.60) | 0.56 (0.52 to 0.61) | 0.58 (0.53 to 0.63) | 0.62 (0.57 to 0.68) |
| Neurotic disorder (N=8,356) | 0.55 (0.49 to 0.62) | 0.58 (0.52 to 0.65) | 0.60 (0.53 to 0.67) | 0.58 (0.52 to 0.66) | 0.67 (0.60 to 0.75) | 0.77 (0.68 to 0.87) |
| Schizophrenia (N=4,676) | 0.59 (0.47 to 0.74) | 0.77 (0.61 to 0.96) | 0.83 (0.65 to 1.05) | 0.83 (0.65 to 1.05) | 0.87 (0.68 to 1.12) | 0.96 (0.75 to 1.23) |
| Anti-depressants (N=56,756) | 0.63 (0.60 to 0.66) | 0.62 (0.59 to 0.65) | 0.63 (0.60 to 0.66) | 0.63 (0.60 to 0.66) | 0.67 (0.64 to 0.71) | 0.71 (0.67 to 0.75) |
| Anti-psychotics (N=11,748) | 0.63 (0.56 to 0.70) | 0.70 (0.62 to 0.79) | 0.74 (0.65 to 0.83) | 0.76 (0.67 to 0.86) | 0.83 (0.73 to 0.94) | 0.9 (0.79 to 1.02) |
| Hypnotics/anxiolytics (N=31,291) | 0.62 (0.58 to 0.66) | 0.65 (0.61 to 0.69) | 0.67 (0.63 to 0.72) | 0.69 (0.65 to 0.74) | 0.76 (0.71 to 0.81) | 0.83 (0.77 to 0.88) |
| Mood stabilisers (N=4,728) | 0.90 (0.73 to 1.11) | 0.93 (0.75 to 1.15) | 0.96 (0.77 to 1.18) | 1.00 (0.81 to 1.23) | 1.01 (0.81 to 1.24) | 0.97 (0.78 to 1.20) |
| ‡ Fully adjusted models were adjusted for: all baseline covariates. Models were estimated using cluster robust standard errors to account for potential clustering of patients between practices. | | | | | | |

eTable 11 presents fully adjusted odds ratios and 95% confidence intervals for the relative association between prescription of varenicline compared to NRT and antidepressant prescription, stratified by mental disorder. There was no association between varenicline and anti-depressant prescription in patients with no mental disorder or patients prescribed antipsychotics at 4-years, in patients with bipolar or patients prescribed mood stabilisers at any follow-up, and patients with schizophrenia at 9-months and 1, 2 and 4-years. Varenicline was associated with lower odds of antidepressant prescription for remaining groups/follow-ups. There was no evidence for an association between varenicline and increased odds of antidepressant prescription.

## eTable 12. Stratified by mental disorder: Fully adjusted odds ratios and 95% confidence intervals for the association between prescription of varenicline versus NRT and hypnotic/anxiolytic prescription at 3, 6 and 9-months, and 1, 2, and 4-years after prescription

| **Logistic regression**  **Fully adjusted odds ratio (95% confidence interval) ‡** | | | | | | |
| --- | --- | --- | --- | --- | --- | --- |
|  | **3-months** | **6-months** | **9-months** | **1-year** | **2-years** | **4-years** |
| No mental disorder (N=156,857) | 0.77 (0.71 to 0.85) | 0.89 (0.83 to 0.94) | 0.93 (0.88 to 0.98) | 0.95 (0.90 to 0.99) | 1.00 (0.96 to 1.04) | 1.05 (1.02 to 1.09) |
| Any mental disorder (N=78,457) | 0.82 (0.78 to 0.86) | 0.84 (0.81 to 0.88) | 0.88 (0.84 to 0.92) | 0.89 (0.86 to 0.93) | 0.93 (0.90 to 0.97) | 0.96 (0.92 to 1.00) |
| Bipolar (N=2,004) | 0.64 (0.44 to 0.91) | 0.64 (0.45 to 0.90) | 0.67 (0.47 to 0.94) | 0.68 (0.48 to 0.95) | 0.74 (0.53 to 1.03) | 0.91 (0.65 to 1.28) |
| Depression (N=17,168) | 0.81 (0.72 to 0.91) | 0.82 (0.73 to 0.91) | 0.90 (0.81 to 0.99) | 0.88 (0.80 to 0.97) | 0.93 (0.85 to 1.01) | 0.94 (0.87 to 1.02) |
| Neurotic disorder (N=8,356) | 0.77 (0.66 to 0.90) | 0.83 (0.72 to 0.95) | 0.89 (0.78 to 1.01) | 0.89 (0.79 to 1.00) | 0.90 (0.80 to 1.01) | 0.96 (0.86 to 1.08) |
| Schizophrenia (N=4,676) | 0.74 (0.57 to 0.95) | 0.76 (0.59 to 0.97) | 0.73 (0.57 to 0.93) | 0.77 (0.60 to 0.98) | 0.81 (0.64 to 1.02) | 0.84 (0.67 to 1.06) |
| Anti-depressants (N=56,756) | 0.81 (0.76 to 0.86) | 0.84 (0.79 to 0.89) | 0.88 (0.83 to 0.93) | 0.89 (0.85 to 0.94) | 0.95 (0.91 to 1.00) | 0.97 (0.93 to 1.02) |
| Anti-psychotics (N=11,748) | 0.67 (0.59 to 0.77) | 0.71 (0.63 to 0.81) | 0.73 (0.65 to 0.83) | 0.76 (0.67 to 0.85) | 0.77 (0.69 to 0.86) | 0.83 (0.74 to 0.93) |
| Hypnotics/anxiolytics (N=31,291) | 0.81 (0.76 to 0.86) | 0.83 (0.78 to 0.88) | 0.86 (0.81 to 0.91) | 0.87 (0.83 to 0.93) | 0.88 (0.83 to 0.93) | 0.89 (0.84 to 0.95) |
| Mood stabilisers (N=4,728) | 0.80 (0.64 to 1.01) | 0.83 (0.67 to 1.02) | 0.85 (0.69 to 1.05) | 0.82 (0.66 to 1.01) | 0.86 (0.71 to 1.04) | 0.90 (0.75 to 1.10) |
| ‡ Fully adjusted models were adjusted for: all baseline covariates. Models were estimated using cluster robust standard errors to account for potential clustering of patients between practices. | | | | | | |

eTable 12 presents fully adjusted odds ratios and 95% confidence intervals for the relative association between prescription of varenicline compared to NRT and hypnotic/anxiolytic prescription, stratified by mental disorder. There was no association between varenicline and hypnotic/anxiolytic prescription in patients with no disorder, bipolar, depression, schizophrenia or in patients prescribed anti-depressants at 2 and 4-years, patients with neurotic disorder at 9-months and at 1, 2 and 4-years, and in patients prescribed mood stabilisers at 3, 6, 9-months and 1, 2 and 4-years. Varenicline was associated with lower odds of hypnotic/anxiolytic prescription for remaining groups/follow-ups. There was no evidence for an association between varenicline and increased odds of hypnotic/anxiolytic prescription.

## eTable 13. Stratified by mental disorder: Propensity score matched logistic regression odds ratios and 95% confidence intervals for the association between prescription of varenicline versus NRT and depression at 3, 6 and 9-months, and 1, 2, and 4-years follow-up

| **Propensity score matched logistic regression**  **Adjusted odds ratio (95% confidence interval) ‡*** | | | | | | |
| --- | --- | --- | --- | --- | --- | --- |
|  | **3-months** | **6-months** | **9-months** | **1-year** | **2-years** | **4-years** |
| No mental disorder (N=112,910) | 0.72 (0.63 to 0.81) | 0.88 (0.80 to 0.96) | 0.90 (0.84 to 0.97) | 0.90 (0.84 to 0.96) | 0.91 (0.86 to 0.96) | 0.95 (0.91 to 0.99) |
| Any mental disorder (N= 30,245) | 0.93 (0.79 to 1.10) | 0.97 (0.86 to 1.10) | 0.98 (0.88 to 1.09) | 1.00 (0.90 to 1.10) | 1.04 (0.97 to 1.13) | 1.07 (1.01 to 1.14) |
| Bipolar (N=357) | 1.52 (0.33 to 6.94) | 1.48 (0.48 to 4.56) | 0.88 (0.34 to 2.29) | 0.81 (0.34 to 1.93) | 1.12 (0.56 to 2.25) | 1.22 (0.65 to 2.30) |
| Neurotic disorder (N= 2,497) | 0.85 (0.51 to 1.42) | 0.98 (0.67 to 1.42) | 0.98 (0.71 to 1.35) | 1.02 (0.76 to 1.36) | 1.10 (0.87 to 1.41) | 1.15 (0.93 to 1.41) |
| Schizophrenia (N=752) | 1.49 (0.41 to 5.48) | 1.32 (0.51 to 3.46) | 1.41 (0.67 to 2.98) | 1.67 (0.86 to 3.25) | 1.64 (0.99 to 2.70) | 1.59 (1.01 to 2.50) |
| Antidepressants (N= 19,571) | 0.92 (0.76 to 1.12) | 0.98 (0.85 to 1.13) | 0.96 (0.84 to 1.10) | 0.99 (0.88 to 1.11) | 1.04 (0.95 to 1.14) | 1.07 (0.98 to 1.16) |
| Antipsychotics (N=3,457) | 0.78 (0.43 to 1.41) | 1.05 (0.69 to 1.59) | 1.11 (0.80 to 1.54) | 1.05 (0.78 to 1.42) | 1.09 (0.83 to 1.43) | 1.10 (0.88 to 1.39) |
| Hypnotics/anxiolytics (N=12,799) | 0.96 (0.74 to 1.24) | 1.00 (0.84 to 1.21) | 1.02 (0.88 to 1.19) | 1.02 (0.90 to 1.17) | 1.03 (0.92 to 1.16) | 1.08 (0.98 to 1.19) |
| Mood stabilisers (N=1,146) | 1.53 (0.58 to 4.00) | 1.31 (0.53 to 3.20) | 1.28 (0.54 to 3.01) | 1.18 (0.61 to 2.29) | 1.35 (0.87 to 2.11) | 1.31 (0.92 to 1.86) |
| ‡Estimates were adjusted for propensity score, and propensity score. Models were estimated using cluster robust standard errors to account for potential clustering of patients between practices. *Patients with depression at baseline were excluded as change depression diagnoses are not regularly recorded in the CPRD. | | | | | | |

eTable 13 presents propensity score matched odds ratios and 95% confidence intervals for the relative association between prescription of varenicline compared to NRT, and depression, stratified by mental disorder. Varenicline was associated with lower odds of depression in patients with no mental disorder at 3, 6, 9-months and 1 and 2 -years follow-up. Varenicline was associated with increased odds of depression in people with any mental disorder at 4-years follow-up. There was no association between varenicline and depression in all other mental health groups at all other follow-ups.

## eTable 14. Stratified by mental disorder: Propensity score matched logistic regression odds ratios and 95% confidence intervals for the association between prescription of varenicline versus NRT and neurotic disorder at 3, 6 and 9-months, and 1, 2, and 4-years follow-up

| **Propensity score matched logistic regression**  **Adjusted odds ratio (95% confidence interval) ‡*** | | | | | | |
| --- | --- | --- | --- | --- | --- | --- |
|  | **3-months** | **6-months** | **9-months** | **1-year** | **2-years** | **4-years** |
| No mental disorder (N=112,910) | 0.73 (0.62 to 0.87) | 0.87 (0.76 to 0.99) | 0.84 (0.75 to 0.93) | 0.85 (0.78 to 0.94) | 0.87 (0.81 to 0.94) | 0.91 (0.86 to 0.97) |
| Any mental disorder (N=34,161) | 0.87 (0.69 to 1.10) | 0.93 (0.79 to 1.08) | 0.96 (0.84 to 1.10) | 0.95 (0.85 to 1.07) | 0.98 (0.89 to 1.07) | 0.99 (0.91 to 1.07) |
| Bipolar (N=385) | Cannot estimate | Cannot estimate | 0.29 (0.04 to 2.34) | 0.48 (0.11 to 2.09) | 0.60 (0.20 to 1.80) | 0.66 (0.25 to 1.76) |
| Depression (N=6,131) | 0.71 (0.44 to 1.16) | 0.86 (0.61 to 1.21) | 0.92 (0.69 to 1.22) | 0.95 (0.75 to 1.20) | 0.97 (0.79 to 1.20) | 0.99 (0.83 to 1.18) |
| Schizophrenia (N=802) | 1.43 (0.31 to 6.64) | 1.53 (0.54 to 4.37) | 1.09 (0.45 to 2.61) | 1.06 (0.51 to 2.19) | 1.20 (0.66 to 2.19) | 0.93 (0.58 to 1.49) |
| Antidepressants (N=23,737) | 0.91 (0.71 to 1.18) | 0.94 (0.79 to 1.13) | 1.00 (0.85 to 1.16) | 0.97 (0.85 to 1.11) | 1.00 (0.89 to 1.11) | 1.00 (0.91 to 1.09) |
| Antipsychotics (N=3,644) | 0.89 (0.46 to 1.73) | 1.04 (0.64 to 1.67) | 1.01 (0.68 to 1.50) | 1.06 (0.74 to 1.53) | 0.97 (0.72 to 1.30) | 0.97 (0.76 to 1.23) |
| Hypnotics/anxiolytics (N=13,529) | 0.77 (0.55 to 1.06) | 0.81 (0.64 to 1.01) | 0.86 (0.71 to 1.05) | 0.87 (0.73 to 1.04) | 0.90 (0.79 to 1.03) | 0.92 (0.82 to 1.03) |
| Mood stabilisers (N=1,224) | 1.10 (0.35 to 3.51) | 1.04 (0.41 to 2.67) | 0.93 (0.43 to 2.00) | 0.67 (0.33 to 1.34) | 0.70 (0.42 to 1.17) | 0.91 (0.60 to 1.39) |
| ‡Estimates were adjusted for propensity score, and propensity score. Models were estimated using cluster robust standard errors to account for potential clustering of patients between practices. *Patients with a neurotic disorder diagnosis at baseline were excluded as change neurotic disorder diagnoses are not regularly recorded in the CPRD. | | | | | | |

eTable 14 presents propensity score matched odds ratios and 95% confidence intervals for the relative association between prescription of varenicline compared to NRT, and neurotic disorder, stratified by mental disorder. Varenicline was associated with lower odds of neurotic disorder in patients with no mental disorder at 3, 6 and 9-months, and 1, 2 and 4-years follow-up. There was no association between varenicline and neurotic disorder in all other mental health groups at all other follow-ups. There was no evidence for an association between varenicline and an increased odds of neurotic disorder.

## eTable 15. Stratified by mental disorder: Propensity score matched logistic regression odds ratios and 95% confidence intervals for the association between prescription of varenicline versus NRT and antidepressant prescription at 3, 6 and 9-months, and 1, 2, and 4-years follow-up

| **Propensity score matched logistic regression**  **Adjusted odds ratio (95% confidence interval) †** | | | | | | |
| --- | --- | --- | --- | --- | --- | --- |
|  | **3-months** | **6-months** | **9-months** | **1-year** | **2-years** | **4-years** |
| No mental disorder (N=112,910) | 0.69 (0.63 to 0.75) | 0.82 (0.77 to 0.87) | 0.87 (0.82 to 0.91) | 0.88 (0.84 to 0.92) | 0.93 (0.90 to 0.97) | 0.99 (0.96 to 1.02) |
| Any mental disorder (N=38,220) | 0.67 (0.64 to 0.70) | 0.69 (0.65 to 0.72) | 0.70 (0.67 to 0.74) | 0.71 (0.68 to 0.75) | 0.76 (0.73 to 0.80) | 0.81 (0.77 to 0.85) |
| Bipolar (N=418) | 0.81 (0.49 to 1.33) | 0.88 (0.53 to 1.45) | 0.87 (0.52 to 1.45) | 0.84 (0.50 to 1.42) | 0.86 (0.50 to 1.49) | 0.92 (0.53 to 1.59) |
| Depression (N=7,484) | 0.58 (0.52 to 0.65) | 0.60 (0.53 to 0.67) | 0.59 (0.53 to 0.66) | 0.59 (0.53 to 0.67) | 0.61 (0.54 to 0.68) | 0.65 (0.58 to 0.73) |
| Neurotic disorder (N=3,874) | 0.60 (0.52 to 0.70) | 0.64 (0.55 to 0.74) | 0.66 (0.56 to 0.76) | 0.65 (0.56 to 0.75) | 0.72 (0.61 to 0.85) | 0.80 (0.69 to 0.94) |
| Schizophrenia (N=876) | 0.67 (0.48 to 0.94) | 0.83 (0.59 to 1.15) | 0.87 (0.63 to 1.21) | 0.87 (0.63 to 1.21) | 0.90 (0.65 to 1.25) | 0.96 (0.70 to 1.32) |
| Antidepressants (N= 26,330) | 0.65 (0.61 to 0.69) | 0.64 (0.60 to 0.69) | 0.65 (0.61 to 0.70) | 0.65 (0.60 to 0.70) | 0.68 (0.63 to 0.74) | 0.71 (0.65 to 0.78) |
| Antipsychotics (N=3,970) | 0.71 (0.61 to 0.83) | 0.78 (0.67 to 0.91) | 0.81 (0.70 to 0.94) | 0.83 (0.72 to 0.96) | 0.88 (0.76 to 1.02) | 0.94 (0.81 to 1.10) |
| Hypnotics/anxiolytics (N=15,270) | 0.67 (0.62 to 0.73) | 0.70 (0.65 to 0.76) | 0.72 (0.67 to 0.78) | 0.74 (0.68 to 0.80) | 0.79 (0.73 to 0.86) | 0.85 (0.78 to 0.93) |
| Mood stabilisers (N=1,284) | 0.90 (0.69 to 1.19) | 0.92 (0.70 to 1.19) | 0.94 (0.72 to 1.24) | 0.97 (0.74 to 1.28) | 0.99 (0.75 to 1.31) | 0.96 (0.73 to 1.27) |
| ‡Estimates were adjusted for propensity score, and propensity score. Models were estimated using cluster robust standard errors to account for potential clustering of patients between practices. | | | | | | |

eTable 15 presents propensity score matched odds ratios and 95% confidence intervals for the relative association between prescription of varenicline compared to NRT, and antidepressant prescription, stratified by mental disorder. There was no association between varenicline and antidepressant prescription in patients with no mental disorder at 4-years, in patients with bipolar or schizophrenia at any follow-up, in patients prescribed antipsychotics at 2 and 4-years, or patients prescribed mood stabilisers at any follow-up. Varenicline was associated with lower odds of antidepressant prescription for remaining groups/follow-ups. There was no evidence for an association between varenicline and increased odds of antidepressant prescription.

## eTable 16. Stratified by mental disorder: Propensity score matched logistic regression odds ratios and 95% confidence intervals for the association between prescription of varenicline versus NRT and hypnotic/anxiolytic prescription at 3, 6 and 9-months, and 1, 2, and 4-years follow-up

| **Propensity score matched logistic regression**  **Adjusted odds ratio (95% confidence interval) †** | | | | | | |
| --- | --- | --- | --- | --- | --- | --- |
|  | **3-months** | **6-months** | **9-months** | **1-year** | **2-years** | **4-years** |
| No mental disorder (N=112,910) | 0.77 (0.70 to 0.86) | 0.89 (0.82 to 0.96) | 0.93 (0.87 to 0.99) | 0.94 (0.89 to 1.00) | 0.99 (0.94 to 1.03) | 1.03 (1.00 to 1.08) |
| Any mental disorder (N=38,220) | 0.83 (0.78 to 0.89) | 0.86 (0.81 to 0.91) | 0.89 (0.84 to 0.94) | 0.90 (0.85 to 0.95) | 0.94 (0.89 to 0.99) | 0.96 (0.91 to 1.01) |
| Bipolar (N=418) | 0.72 (0.44 to 1.16) | 0.71 (0.44 to 1.13) | 0.71 (0.45 to 1.14) | 0.73 (0.45 to 1.19) | 0.79 (0.50 to 1.24) | 0.94 (0.59 to 1.50) |
| Depression (N=7,484) | 0.83 (0.71 to 0.97) | 0.83 (0.73 to 0.96) | 0.91 (0.80 to 1.03) | 0.89 (0.79 to 1.01) | 0.94 (0.84 to 1.05) | 0.96 (0.86 to 1.07) |
| Neurotic disorder (N=3,874) | 0.78 (0.64 to 0.96) | 0.82 (0.68 to 0.99) | 0.88 (0.74 to 1.04) | 0.88 (0.74 to 1.04) | 0.89 (0.76 to 1.05) | 0.95 (0.81 to 1.10) |
| Schizophrenia (N=876) | 0.75 (0.52 to 1.08) | 0.78 (0.55 to 1.11) | 0.76 (0.54 to 1.08) | 0.80 (0.57 to 1.12) | 0.82 (0.59 to 1.14) | 0.85 (0.62 to 1.16) |
| Antidepressants (N= 26,330) | 0.82 (0.76 to 0.89) | 0.85 (0.80 to 0.91) | 0.89 (0.83 to 0.95) | 0.90 (0.84 to 0.96) | 0.95 (0.89 to 1.01) | 0.97 (0.92 to 1.03) |
| Antipsychotics (N=3,970) | 0.75 (0.62 to 0.90) | 0.78 (0.66 to 0.93) | 0.81 (0.68 to 0.95) | 0.82 (0.69 to 0.97) | 0.83 (0.71 to 0.97) | 0.88 (0.76 to 1.03) |
| Hypnotics/anxiolytics (N=15,270) | 0.82 (0.76 to 0.89) | 0.83 (0.77 to 0.90) | 0.86 (0.80 to 0.93) | 0.88 (0.81 to 0.95) | 0.87 (0.81 to 0.95) | 0.89 (0.81 to 0.97) |
| Mood stabilisers (N=1,284) | 0.83 (0.61 to 1.13) | 0.86 (0.64 to 1.15) | 0.89 (0.67 to 1.18) | 0.85 (0.64 to 1.14) | 0.88 (0.67 to 1.16) | 0.92 (0.69 to 1.22) |
| ‡Estimates were adjusted for propensity score, and propensity score. Models were estimated using cluster robust standard errors to account for potential clustering of patients between practices. | | | | | | |

eTable 16 propensity score matched odds ratios and 95% confidence intervals for the relative association between prescription of varenicline compared to NRT, and hypnotic/anxiolytic prescription, stratified by mental disorder. There was no association between varenicline and hypnotic/anxiolytic prescription for most patients at most follow-ups. Varenicline was associated with lower odds of hypnotic/anxiolytic prescription in patients with no mental disorder at 3, 6 and 9-months, and in patients with any mental disorder at 3, 6 and 9-months and at 1 and 2-years, in patients with depression at 3 and 6-months, patients prescribed anti-depressants at 3, 6 and 9-months and 1-year, and in patients prescribed hypnotic/anxiolytics at all follow-ups. There was an association between varenicline and increased odds of hypnotic/anxiolytic prescription at 4-years in patients with no mental disorder.

## eTable 17. Stratified by mental disorder: Linear regression risk difference per 100 patients and 95% confidence intervals for the association between prescription of varenicline versus NRT and depression at 3, 6 and 9-months, and 1, 2, and 4-years follow-up

| **Linear regression**  **Risk difference per 100 patients (95% confidence interval) ‡*** | | | | | | |
| --- | --- | --- | --- | --- | --- | --- |
|  | **3-months** | **6-months** | **9-months** | **1-year** | **2-years** | **4-years** |
| No mental disorder (N=136,654) | -0.30 (-0.40 to -0.19) | -0.24 (-0.39 to -0.08) | -0.29 (-0.48 to -0.11) | -0.39 (-0.61 to -0.17) | -0.65 (-0.94 to -0.35) | -0.61 (-0.99 to -0.24) |
| Any mental disorder (N=53,883) | -0.16 (-0.47 to 0.14) | -0.22 (-0.66 to 0.21) | -0.20 (-0.72 to 0.32) | -0.12 (-0.71 to 0.46) | 0.31 (-0.44 to 1.06) | 0.66 (-0.23 to 1.55) |
| Bipolar (N=1,534) | 1.56 (-1.29 to 4.41) | 2.03 (-1.63 to 5.68) | 0.51 (-3.35 to 4.37) | 0.32 (-3.88 to 4.51) | 3.23 (-2.39 to 8.86) | 4.92 (-1.67 to 11.51) |
| Neurotic disorder (N=4,569) | -0.94 (-2.18 to 0.29) | -1.13 (-2.85 to 0.58) | -1.56 (-3.50 to 0.38) | -1.33 (-3.43 to 0.78) | -1.22 (-3.78 to 1.34) | -0.93 (-3.86 to 1.99) |
| Schizophrenia (N=3,646) | 1.03 (-0.70 to 2.76) | 1.35 (-0.86 to 3.57) | 2.40 (-0.40 to 5.20) | 4.51 (1.27 to 7.75) | 7.25 (3.07 to 11.43) | 9.10 (4.58 to 13.62) |
| Anti-depressants (N=36,702) | -0.15 (-0.55 to 0.25) | -0.16 (-0.72 to 0.40) | -0.28 (-0.96 to 0.40) | -0.12 (-0.90 to 0.65) | 0.43 (-0.55 to 1.41) | 0.61 (-0.54 to 1.76) |
| Anti-psychotics (N=8,970) | -0.19 (-0.94 to 0.56) | 0.45 (-0.70 to 1.60) | 0.87 (-0.51 to 2.25) | 0.70 (-0.81 to 2.22) | 1.01 (-0.90 to 2.91) | 1.30 (-0.88 to 3.47) |
| Hypnotics/anxiolytics (N=22,513) | -0.03 (-0.52 to 0.46) | -0.08 (-0.75 to 0.60) | -0.06 (-0.85 to 0.73) | -0.12 (-1.00 to 0.75) | -0.09 (-1.18 to 1.00) | 0.40 (-0.87 to 1.67) |
| Mood stabilisers (N=3,689) | 0.59 (-0.69 to 1.86) | 0.46 (-1.15 to 2.07) | 0.84 (-1.13 to 2.80) | 0.88 (-1.33 to 3.09) | 3.20 (0.14 to 6.26) | 4.33 (0.55 to 8.10) |
| ‡Partially adjusted estimates were adjusted for age, sex, year of prescription. Models were estimated using cluster robust standard errors to account for potential clustering of patients between practices. *Patients with a depression diagnosis within 365 days of baseline were excluded as change depression diagnoses are not regularly recorded in the CPRD. | | | | | | |

eTable 17 presents risk differences per 100 patients treated and 95% confidence intervals for the relative association between prescription of varenicline compared to NRT and depression, stratified by mental disorder. Varenicline was associated with increased risk of depression in patients with bipolar at 2 and 4-years, patients with schizophrenia at 1, 2 and 4-years, and in patients prescribed mood stabilisers at 2 and 4-years follow-up. Varenicline was associated with decreased risk of depression in those with no mental disorder at 3, 6 and 9-months and at 1, 2 and 4-years follow-up. There was no association between varenicline and depression in all other patient groups at all other follow-ups.

## eTable 18. Stratified by mental disorder: Linear regression risk difference per 100 patients and 95% confidence intervals for the association between prescription of varenicline versus NRT and neurotic disorder at 3, 6 and 9-months, and 1, 2, and 4-years follow-up

| **Linear regression**  **Risk difference per 100 patients (95% confidence interval) ‡*** | | | | | | |
| --- | --- | --- | --- | --- | --- | --- |
|  | **3-months** | **6-months** | **9-months** | **1-year** | **2-years** | **4-years** |
| No mental disorder (N=136,654) | -0.14 (-0.22 to -0.06) | -0.12 (-0.24 to 0.00) | -0.26 (-0.40 to -0.12) | -0.29 (-0.46 to -0.13) | -0.46 (-0.68 to -0.24) | -0.51 (-0.79 to -0.22) |
| Any mental disorder (N=61,309) | -0.19 (-0.39 to 0.00) | -0.17 (-0.46 to 0.12) | -0.15 (-0.50 to 0.20) | -0.32 (-0.72 to 0.07) | -0.39 (-0.92 to 0.14) | -0.48 (-1.13 to 0.18) |
| Bipolar (N=1,615) | -1.59 (-2.26 to -0.92) | -1.96 (-3.30 to -0.61) | -2.16 (-3.95 to -0.38) | -2.08 (-4.48 to 0.32) | -2.83 (-6.11 to 0.45) | -3.24 (-7.42 to 0.94) |
| Depression (N=11,995) | -0.47 (-0.97 to 0.03) | -0.60 (-1.34 to 0.14) | -0.49 (-1.41 to 0.42) | -0.45 (-1.46 to 0.57) | -0.9 (-2.19 to 0.39) | -0.99 (-2.54 to 0.57) |
| Schizophrenia (N=3,851) | 0.41 (-0.90 to 1.71) | 0.76 (-1.11 to 2.62) | -0.06 (-2.03 to 1.90) | -0.05 (-2.30 to 2.20) | 1.49 (-1.43 to 4.41) | 0.55 (-2.75 to 3.86) |
| Anti-depressants (N=44,648) | -0.16 (-0.39 to 0.08) | -0.18 (-0.52 to 0.17) | -0.12 (-0.54 to 0.30) | -0.29 (-0.76 to 0.18) | -0.38 (-1.02 to 0.26) | -0.48 (-1.24 to 0.28) |
| Anti-psychotics (N=9,471) | -0.27 (-0.85 to 0.31) | 0.02 (-0.87 to 0.90) | -0.05 (-1.09 to 1.00) | -0.15 (-1.32 to 1.01) | -0.65 (-2.10 to 0.81) | -0.99 (-2.76 to 0.79) |
| Hypnotics/anxiolytics (N=23,966) | -0.46 (-0.79 to -0.13) | -0.77 (-1.26 to -0.29) | -0.79 (-1.39 to -0.19) | -0.99 (-1.66 to -0.31) | -1.34 (-2.20 to -0.49) | -1.55 (-2.60 to -0.50) |
| Mood stabilisers (N=3,890) | 0.19 (-0.90 to 1.27) | -0.09 (-1.37 to 1.19) | -0.23 (-1.70 to 1.24) | -1.35 (-2.85 to 0.15) | -2.55 (-4.54 to -0.56) | -1.64 (-4.27 to 0.98) |
| ‡Partially adjusted estimates were adjusted for age, sex, year of prescription. Models were estimated using cluster robust standard errors to account for potential clustering of patients between practices. *Patients with neurotic disorder within 365 days of baseline were excluded as change neurotic disorder diagnoses are not regularly recorded in the CPRD. | | | | | | |

eTable 18 presents risk differences per 100 patients treated and 95% confidence intervals for the relative association between prescription of varenicline compared to NRT and neurotic disorder, stratified by mental disorder. Varenicline was associated with reduced risk of neurotic disorder in patients with no mental disorder at 3 and 9-months, and at 1, 2 and 4-years follow-up, in patients with bipolar at 3, 6, and 9-months follow-up, in patients prescribed hypnotics/anxiolytics at 3, 6 and 9 months, and 1, 2 and 4-years follow-up, and in patients prescribed mood stabilisers at 2-years follow-up. There was no association between varenicline and neurotic disorder in all other patient groups at all other follow-ups. There was no evidence for an association between varenicline and increased risk of neurotic disorder.

## eTable 19. Stratified by mental disorder: Linear regression risk difference per 100 patients and 95% confidence intervals for the association between prescription of varenicline versus NRT and antidepressant prescription at 3, 6 and 9-months, and 1, 2, and 4-years

| **Linear regression**  **Risk difference per 100 patients (95% confidence interval) ‡** | | | | | | |
| --- | --- | --- | --- | --- | --- | --- |
|  | **3-months** | **6-months** | **9-months** | **1-year** | **2-years** | **4-years** |
| No mental disorder (N=136,654) | -0.90 (-1.07 to -0.73) | -1.03 (-1.27 to -0.79) | -1.12 (-1.40 to -0.84) | -1.33 (-1.65 to -1.01) | -1.40 (-1.81 to -0.99) | -1.11 (-1.61 to -0.62) |
| Any mental disorder (N=68,578) | -12.62 (-13.52 to -11.72) | -11.95 (-12.86 to -11.05) | -11.11 (-12.02 to -10.21) | -10.59 (-11.49 to -9.70) | -8.40 (-9.29 to -7.52) | -6.37 (-7.20 to -5.53) |
| Bipolar (N=1,735) | -6.72 (-14.12 to 0.68) | -4.33 (-11.68 to 3.02) | -4.45 (-11.79 to 2.88) | -4.39 (-11.72 to 2.94) | -2.64 (-9.86 to 4.57) | -0.32 (-7.31 to 6.67) |
| Depression (N=14,695) | -15.44 (-17.42 to -13.46) | -14.37 (-16.35 to -12.39) | -14.02 (-15.96 to -12.08) | -13.18 (-15.10 to -11.27) | -11.49 (-13.29 to -9.70) | -8.96 (-10.60 to -7.33) |
| Neurotic disorder (N=7,269) | -17.00 (-19.61 to -14.38) | -16.03 (-18.68 to -13.37) | -15.57 (-18.24 to -12.90) | -15.74 (-18.41 to -13.06) | -12.58 (-15.24 to -9.91) | -9.76 (-12.29 to -7.24) |
| Schizophrenia (N=4,125) | -9.18 (-14.09 to -4.27) | -3.89 (-8.88 to 1.10) | -2.14 (-7.25 to 2.97) | -1.69 (-6.82 to 3.44) | -0.16 (-5.23 to 4.90) | 1.64 (-3.30 to 6.58) |
| Anti-depressants (N=49,763) | -12.78 (-13.81 to -11.74) | -11.97 (-12.97 to -10.97) | -11.08 (-12.07 to -10.10) | -10.47 (-11.43 to -9.52) | -8.20 (-9.09 to -7.30) | -6.35 (-7.17 to -5.54) |
| Anti-psychotics (N=10,345) | -14.02 (-16.49 to -11.55) | -11.76 (-14.27 to -9.25) | -10.53 (-13.09 to -7.98) | -9.74 (-12.30 to -7.18) | -7.67 (-10.19 to -5.16) | -5.47 (-7.90 to -3.05) |
| Hypnotics/anxiolytics (N=27,214) | -15.58 (-16.99 to -14.18) | -14.77 (-16.20 to -13.35) | -14.16 (-15.60 to -12.72) | -13.5 (-14.92 to -12.09) | -11.17 (-12.55 to -9.79) | -8.82 (-10.15 to -7.49) |
| Mood stabilisers (N=4,104) | -4.36 (-8.91 to 0.19) | -3.15 (-7.68 to 1.39) | -2.14 (-6.72 to 2.45) | -1.14 (-5.71 to 3.43) | -0.94 (-5.33 to 3.45) | -0.98 (-5.28 to 3.31) |
| ‡Partially adjusted estimates were adjusted for age, sex, year of prescription. Models were estimated using cluster robust standard errors to account for potential clustering of patients between practices. | | | | | | |

eTable 19 presents risk differences per 100 patients treated and 95% confidence intervals for the relative association between prescription of varenicline compared to NRT and antidepressant prescription, stratified by mental disorder. There was no association between varenicline and anti-depressant prescription in patients with bipolar at any follow-up, or schizophrenia at 6 and 9-months and at 1, 2 and 4-years follow-up, and no association in patients prescribed mood stabilisers at all follow-ups. Varenicline was associated with reduced risk of antidepressant prescription for remaining groups/follow-ups. There was no evidence for an association between varenicline and increased risk of antidepressant prescription.

## eTable 20. Stratified by mental disorder: Linear regression risk difference per 100 patients and 95% confidence intervals for the association between prescription of varenicline versus NRT and hypnotic/anxiolytic prescription at 3, 6 and 9-months, and 1, 2, and 4-years

| **Linear regression**  **Risk difference per 100 patients (95% confidence interval) ‡** | | | | | | |
| --- | --- | --- | --- | --- | --- | --- |
|  | **3-months** | **6-months** | **9-months** | **1-year** | **2-years** | **4-years** |
| No mental disorder (N=136,654) | -0.49 (-0.63 to -0.35) | -0.50 (-0.69 to -0.31) | -0.52 (-0.75 to -0.29) | -0.61 (-0.87 to -0.35) | -0.53 (-0.87 to -0.19) | -0.22 (-0.65 to 0.21) |
| Any mental disorder (N=68,578) | -4.81 (-5.51 to -4.11) | -4.82 (-5.59 to -4.06) | -4.33 (-5.14 to -3.53) | -4.22 (-5.05 to -3.38) | -3.46 (-4.34 to -2.58) | -2.97 (-3.91 to -2.04) |
| Bipolar (N=1,735) | -6.84 (-13.44 to -0.25) | -7.16 (-13.96 to -0.36) | -6.74 (-13.94 to 0.46) | -6.66 (-13.96 to 0.64) | -5.39 (-12.94 to 2.16) | -0.77 (-8.01 to 6.46) |
| Depression (N=14,695) | -3.85 (-5.17 to -2.53) | -4.57 (-6.04 to -3.10) | -3.57 (-5.17 to -1.97) | -3.91 (-5.58 to -2.23) | -3.61 (-5.41 to -1.82) | -3.51 (-5.38 to -1.63) |
| Neurotic disorder (N=7,269) | -5.83 (-7.89 to -3.77) | -5.97 (-8.23 to -3.71) | -4.83 (-7.23 to -2.42) | -5.16 (-7.60 to -2.71) | -5.26 (-7.85 to -2.67) | -4.62 (-7.32 to -1.92) |
| Schizophrenia (N=4,125) | -5.71 (-10.03 to -1.40) | -5.21 (-9.90 to -0.51) | -5.96 (-10.79 to -1.12) | -5.38 (-10.37 to -0.39) | -4.33 (-9.37 to 0.72) | -3.35 (-8.50 to 1.79) |
| Anti-depressants (N=49,763) | -5.20 (-6.01 to -4.40) | -5.36 (-6.24 to -4.48) | -4.93 (-5.85 to -4.00) | -4.91 (-5.87 to -3.95) | -3.85 (-4.90 to -2.81) | -3.49 (-4.60 to -2.38) |
| Anti-psychotics (N=10,345) | -10.95 (-13.02 to -8.88) | -11.03 (-13.23 to -8.82) | -10.79 (-13.10 to -8.49) | -10.48 (-12.89 to -8.07) | -10.50 (-12.96 to -8.04) | -9.39 (-11.95 to -6.82) |
| Hypnotics/anxiolytics (N=27,214) | -11.10 (-12.48 to -9.72) | -10.73 (-12.12 to -9.33) | -9.74 (-11.14 to -8.34) | -9.24 (-10.63 to -7.85) | -8.53 (-9.89 to -7.16) | -7.51 (-8.81 to -6.21) |
| Mood stabilisers (N=4,104) | -6.98 (-10.75 to -3.21) | -6.72 (-10.68 to -2.75) | -6.50 (-10.63 to -2.37) | -7.35 (-11.61 to -3.09) | -6.62 (-10.96 to -2.28) | -5.74 (-10.14 to -1.34) |
| ‡Partially adjusted estimates were adjusted for age, sex, year of prescription. Models were estimated using cluster robust standard errors to account for potential clustering of patients between practices. | | | | | | |

eTable 20 presents risk differences per 100 patients treated and 95% confidence intervals for the relative association between prescription of varenicline compared to NRT and hypnotic/anxiolytic prescription, stratified by mental disorder. There was no association between varenicline and hypnotic/anxiolytic prescription in patients with no mental disorders at 4-years, patients with bipolar at 9-months, and at 1, 2 and 4-years, patients with schizophrenia at 2 and 4-years. Varenicline was associated with reduced risk of hypnotic/anxiolytic prescription for remaining groups/follow-ups. There was no evidence for an association between varenicline and increased risk of hypnotic/anxiolytic prescription.

## eTable 21. Stratified by mental disorder: Instrumental variable regression risk difference per 100 patients and 95% confidence intervals for the association between prescription of varenicline versus NRT and depression at 3, 6 and 9-months, and 1, 2, and 4-years follow-up

| **Instrumental variable regression‡***  **Risk difference per 100 patients (95% confidence interval)** | | | | | | |
| --- | --- | --- | --- | --- | --- | --- |
|  | **3-months** | **6-months** | **9-months** | **1-year** | **2-years** | **4-years** |
| No mental disorder (N=136,654) | -0.33 (-0.66 to -0.01) | -0.29 (-0.77 to 0.19) | -0.15 (-0.74 to 0.43) | -0.11 (-0.78 to 0.57) | -0.11 (-1.04 to 0.83) | -0.41 (-1.63 to 0.80) |
| Any mental disorder (N=53,883) | -0.29 (-1.25 to 0.67) | 0.29 (-1.08 to 1.66) | 1.07 (-0.64 to 2.79) | 1.80 (-0.18 to 3.77) | 3.12 (0.57 to 5.67) | 3.88 (0.85 to 6.90) |
| Bipolar (N=1,534) | 0.75 (-8.72 to 10.22) | 2.75 (-9.86 to 15.35) | -3.65 (-18.71 to 11.41) | -5.54 (-22.7 to 11.62) | -1.01 (-21.54 to 19.52) | 2.56 (-21.7 to 26.81) |
| Neurotic disorder (N=4,569) | -2.52 (-5.82 to 0.79) | -3.91 (-8.70 to 0.88) | -2.11 (-7.57 to 3.36) | -2.05 (-8.17 to 4.06) | 2.50 (-5.12 to 10.12) | 4.11 (-4.12 to 12.34) |
| Schizophrenia (N=3,646) | 0.58 (-4.80 to 5.97) | 5.79 (-1.78 to 13.35) | 8.80 (-0.14 to 17.75) | 13.29 (3.10 to 23.48) | 10.86 (-2.54 to 24.25) | 19.72 (4.33 to 35.11) |
| Anti-depressants (N=36,702) | -0.46 (-1.75 to 0.83) | -0.05 (-1.90 to 1.80) | 0.61 (-1.65 to 2.88) | 1.22 (-1.41 to 3.85) | 2.29 (-1.00 to 5.57) | 2.92 (-0.92 to 6.75) |
| Anti-psychotics (N=8,970) | -0.52 (-3.14 to 2.10) | 3.56 (-0.41 to 7.52) | 6.12 (1.34 to 10.89) | 5.77 (0.29 to 11.25) | 4.57 (-2.11 to 11.25) | 6.22 (-1.51 to 13.94) |
| Hypnotics/anxiolytics (N=22,513) | 0.20 (-1.25 to 1.66) | 1.45 (-0.63 to 3.53) | 2.69 (0.12 to 5.26) | 3.60 (0.72 to 6.48) | 4.03 (0.41 to 7.64) | 3.67 (-0.49 to 7.83) |
| Mood stabilisers (N=3,689) | 2.91 (-1.76 to 7.59) | 2.74 (-3.28 to 8.77) | 7.28 (-0.03 to 14.58) | 5.25 (-3.28 to 13.79) | 8.55 (-2.26 to 19.37) | 8.95 (-3.55 to 21.44) |
| ‡Partially adjusted estimates were adjusted for age, sex, year of prescription. Models were estimated using cluster robust standard errors to account for potential clustering of patients between practices. * Patients with a depression diagnosis within 365 days of baseline were excluded as change depression diagnoses are not regularly recorded in the CPRD. | | | | | | |

eTable 21 presents risk differences per 100 patients treated and 95% confidence intervals for the relative association between prescription of varenicline compared to NRT and depression, stratified by mental disorder, derived from instrumental variable models. There was evidence for a reduced risk of depression in patients with no mental disorder at 3-months follow-up. There was evidence for an increased risk of depression in patients any mental disorder at 2 and 4-years, in patients with schizophrenia at 1 and 4-years follow-up, in patients prescribed anti-psychotics at 9-months and 1-year follow-up, and in patients prescribed hypnotics/anxiolytics at 9-months, and 1 and 2-yearsfollow-up. There was no association between varenicline and depression for remaining groups/follow-ups.

## eTable 22. Stratified by mental disorder: Instrumental variable regression risk difference per 100 patients and 95% confidence intervals for the association between prescription of varenicline versus NRT and neurotic disorder at 3, 6 and 9-months, and 1, 2, and 4-years follow-up

| **Instrumental variable regression‡***  **Risk difference per 100 patients (95% confidence interval)** | | | | | | |
| --- | --- | --- | --- | --- | --- | --- |
|  | **3-months** | **6-months** | **9-months** | **1-year** | **2-years** | **4-years** |
| No mental disorder (N=136,654) | 0.15 (-0.08 to 0.39) | 0.42 (0.05 to 0.80) | 0.35 (-0.10 to 0.80) | 0.35 (-0.18 to 0.88) | 0.30 (-0.45 to 1.05) | 0.08 (-0.89 to 1.04) |
| Any mental disorder (N=68,578) | -0.35 (-1.01 to 0.30) | -0.12 (-1.08 to 0.84) | 0.44 (-0.76 to 1.63) | 0.33 (-1.04 to 1.70) | 0.67 (-1.18 to 2.52) | 1.15 (-1.16 to 3.46) |
| Bipolar (N=1,615) | -4.16 (-12.71 to 4.39) | -0.57 (-11.67 to 10.54) | 4.53 (-8.07 to 17.13) | 9.96 (-4.10 to 24.02) | 8.61 (-8.98 to 26.2) | 7.92 (-12.44 to 28.28) |
| Depression (N=11,995) | -1.19 (-2.70 to 0.32) | -0.91 (-3.18 to 1.35) | -0.94 (-3.85 to 1.98) | 0.01 (-3.22 to 3.24) | -0.41 (-4.72 to 3.91) | -0.70 (-5.72 to 4.33) |
| Schizophrenia (N=3,851) | 2.06 (-2.89 to 7.02) | 2.52 (-3.84 to 8.89) | 0.13 (-6.97 to 7.23) | 1.99 (-6.27 to 10.25) | 5.44 (-5.00 to 15.89) | 6.17 (-6.10 to 18.44) |
| Anti-depressants (N=44,648) | -0.60 (-1.37 to 0.17) | -0.08 (-1.24 to 1.08) | 0.52 (-0.93 to 1.97) | 0.42 (-1.21 to 2.06) | 0.14 (-2.09 to 2.37) | 0.45 (-2.25 to 3.14) |
| Anti-psychotics (N=9,471) | -0.13 (-2.27 to 2.02) | 1.47 (-1.73 to 4.67) | 2.30 (-1.58 to 6.19) | 3.55 (-0.77 to 7.87) | 3.07 (-2.32 to 8.46) | -0.44 (-6.82 to 5.94) |
| Hypnotics/anxiolytics (N=23,966) | -0.76 (-1.90 to 0.37) | -0.99 (-2.75 to 0.77) | 0.69 (-1.53 to 2.90) | 0.71 (-1.75 to 3.16) | 1.32 (-1.76 to 4.41) | 2.52 (-1.28 to 6.31) |
| Mood stabilisers (N=3,890) | -0.21 (-4.33 to 3.91) | 1.58 (-3.86 to 7.03) | 2.29 (-3.83 to 8.42) | 3.07 (-3.85 to 9.98) | 4.61 (-4.20 to 13.41) | 4.57 (-5.92 to 15.05) |
| ‡Partially adjusted estimates were adjusted for age, sex, year of prescription. Models were estimated using cluster robust standard errors to account for potential clustering of patients between practices. *Patients with a neurotic disorder diagnosis within 365 days of baseline were excluded as change neurotic disorder diagnoses are not regularly recorded in the CPRD. | | | | | | |

eTable 22 presents risk differences per 100 patients treated and 95% confidence intervals for the relative association between prescription of varenicline compared to NRT and neurotic disorder, stratified by mental disorder, derived from instrumental variable models. In patients with no mental disorder there was an increased risk of neurotic disorder at 6-months. There was no evidence for an association between varenicline and neurotic disorder for the remaining groups/follow-ups.

## eTable 23. Stratified by mental disorder: Instrumental variable regression risk difference per 100 patients and 95% confidence intervals for the association between prescription of varenicline versus NRT and antidepressant prescription at 3, 6 and 9-months, and 1, 2, and 4-years follow-up

| **Instrumental variable regression‡**  **Risk difference per 100 patients (95% confidence interval)** | | | | | | |
| --- | --- | --- | --- | --- | --- | --- |
|  | **3-months** | **6-months** | **9-months** | **1-year** | **2-years** | **4-years** |
| No mental disorder (N=136,654) | -0.64 (-1.14 to -0.15) | -0.20 (-0.91 to 0.51) | 0.08 (-0.77 to 0.94) | 0.01 (-0.97 to 0.99) | 0.41 (-0.85 to 1.67) | 0.65 (-0.93 to 2.24) |
| Any mental disorder (N=68,578) | -5.71 (-8.49 to -2.94) | -5.36 (-8.13 to -2.59) | -4.56 (-7.31 to -1.80) | -4.13 (-6.90 to -1.36) | -0.95 (-3.62 to 1.71) | -0.73 (-3.29 to 1.83) |
| Bipolar (N=1,735) | -9.94 (-39.28 to 19.40) | -13.31 (-43.14 to 16.52) | -15.09 (-44.90 to 14.71) | -19.25 (-48.93 to 10.42) | -5.04 (-33.77 to 23.69) | -1.10 (-29.32 to 27.12) |
| Depression (N=14,695) | -3.96 (-9.87 to 1.94) | -4.72 (-10.55 to 1.10) | -5.32 (-10.93 to 0.30) | -4.92 (-10.48 to 0.64) | -3.79 (-8.95 to 1.36) | -3.39 (-8.09 to 1.30) |
| Neurotic disorder (N=7,269) | -9.62 (-17.37 to -1.87) | -10.70 (-18.60 to -2.80) | -10.08 (-17.69 to -2.47) | -8.70 (-16.24 to -1.16) | -3.46 (-10.69 to 3.78) | -3.42 (-10.43 to 3.59) |
| Schizophrenia (N=4,125) | 5.71 (-12.45 to 23.87) | 18.50 (0.09 to 36.91) | 18.56 (0.18 to 36.94) | 18.96 (0.55 to 37.38) | 19.64 (1.52 to 37.75) | 18.13 (0.60 to 35.66) |
| Anti-depressants (N=49,763) | -7.24 (-10.32 to -4.16) | -7.02 (-9.90 to -4.13) | -6.62 (-9.41 to -3.84) | -6.90 (-9.65 to -4.14) | -4.05 (-6.58 to -1.51) | -3.97 (-6.29 to -1.65) |
| Anti-psychotics (N=10,345) | 2.85 (-5.82 to 11.52) | 6.50 (-2.14 to 15.13) | 9.58 (1.07 to 18.09) | 11.07 (2.56 to 19.58) | 11.21 (3.05 to 19.38) | 10.83 (2.85 to 18.81) |
| Hypnotics/anxiolytics (N=27,214) | -7.64 (-12.12 to -3.17) | -6.84 (-11.32 to -2.35) | -6.54 (-11.03 to -2.04) | -6.18 (-10.68 to -1.69) | -2.61 (-6.97 to 1.75) | -2.58 (-6.74 to 1.57) |
| Mood stabilisers (N=4,104) | 4.88 (-11.33 to 21.09) | 6.71 (-9.72 to 23.15) | 8.10 (-8.37 to 24.56) | 9.88 (-6.44 to 26.20) | 13.06 (-2.84 to 28.97) | 14.87 (-0.59 to 30.32) |
| ‡Partially adjusted estimates were adjusted for age, sex, year of prescription. Models were estimated using cluster robust standard errors to account for potential clustering of patients between practices. | | | | | | |

eTable 23 presents risk differences per 100 patients treated and 95% confidence intervals for the relative association between prescription of varenicline compared to NRT and antidepressant prescription, stratified by mental disorder, derived from instrumental variable models. There was evidence for an association between varenicline and reduced risk of anti-depressant prescription in patients with no mental disorder at 3-months follow-up, in patients with any mental disorder at 3, 6, and 9-months and at 1-year follow-up, in patients with neurotic disorder at 3, 6 and 9-months and 1-year follow-up, in patients prescribed anti-depressants at all follow-ups, and in patients prescribed hypnotics/anxiolytics at 3, 6, and 9 months and at 1-year follow-up. There was evidence for an association between varenicline and increased risk of antidepressant prescription in patients with schizophrenia at 6 and 9-months, and at 1, 2, and 4-years follow-up, and in patients prescribed anti-psychotics at 9-months and at 1, 2, and 4 years follow-up. The was no evidence for an association between varenicline and anti-depressant prescription in the remaining groups/follow-ups.

## eTable 24. Stratified by mental disorder: Instrumental variable regression risk difference per 100 patients and 95% confidence intervals for the association between prescription of varenicline versus NRT and hypnotic/anxiolytic prescription at 3, 6 and 9-months, and 1, 2, and 4-years follow-up

| **Instrumental variable regression‡**  **Risk difference per 100 patients (95% confidence interval)** | | | | | | |
| --- | --- | --- | --- | --- | --- | --- |
|  | **3-months** | **6-months** | **9-months** | **1-year** | **2-years** | **4-years** |
| No mental disorder (N=136,654) | 0.14 (-0.26 to 0.54) | 0.28 (-0.29 to 0.84) | 0.07 (-0.63 to 0.76) | 0.00 (-0.80 to 0.81) | -0.17 (-1.23 to 0.90) | 0.47 (-0.89 to 1.83) |
| Any mental disorder (N=68,578) | -1.77 (-4.31 to 0.76) | -1.81 (-4.54 to 0.92) | -0.96 (-3.81 to 1.89) | -0.93 (-3.91 to 2.06) | -0.91 (-4.02 to 2.20) | -1.27 (-4.62 to 2.08) |
| Bipolar (N=1,735) | -29.86 (-57.27 to -2.46) | -31.68 (-59.93 to -3.43) | -24.18 (-52.64 to 4.28) | -19.97 (-48.46 to 8.52) | -25.32 (-54.85 to 4.22) | -19.49 (-48.58 to 9.61) |
| Depression (N=14,695) | -2.73 (-6.86 to 1.41) | -4.29 (-8.94 to 0.36) | -3.12 (-8.08 to 1.83) | -3.82(-9.01 to 1.37) | -3.79 (-9.63 to 2.04) | -6.55 (-12.64 to -0.45) |
| Neurotic disorder (N=7,269) | -5.04 (-11.31 to 1.22) | -7.04 (-13.73 to -0.35) | -5.44 (-12.54 to 1.66) | -5.13 (-12.44 to 2.19) | -5.28 (-12.96 to 2.41) | -5.41 (-13.24 to 2.42) |
| Schizophrenia (N=4,125) | 7.12 (-10.45 to 24.69) | 12.58 (-5.93 to 31.10) | 15.23 (-3.67 to 34.14) | 11.90 (-6.96 to 30.76) | 7.21 (-11.55 to 25.97) | 4.65 (-14.04 to 23.34) |
| Anti-depressants (N=49,763) | -1.43 (-4.35 to 1.50) | -2.03 (-5.16 to 1.10) | -0.99 (-4.26 to 2.28) | -1.41 (-4.83 to 2.00) | -1.20 (-4.80 to 2.40) | -2.18 (-6.06 to 1.70) |
| Anti-psychotics (N=10,345) | 0.41 (-7.61 to 8.43) | 1.03 (-7.24 to 9.31) | 2.51 (-6.07 to 11.09) | 3.63 (-5.19 to 12.45) | -1.68 (-10.51 to 7.14) | 0.66 (-8.21 to 9.53) |
| Hypnotics/anxiolytics (N=27,214) | -3.57 (-8.28 to 1.14) | -2.7 (-7.43 to 2.02) | -2.62 (-7.29 to 2.04) | -0.97 (-5.59 to 3.65) | -2.32 (-6.80 to 2.15) | -2.11 (-6.35 to 2.14) |
| Mood stabilisers (N=4,104) | -1.73 (-15.81 to 12.35) | -3.36 (-18.18 to 11.45) | -1.68 (-17.02 to 13.67) | -2.38 (-18.02 to 13.25) | -2.19 (-17.97 to 13.59) | 0.16 (-15.48 to 15.8) |
| ‡Partially adjusted estimates were adjusted for age, sex, year of prescription. Models were estimated using cluster robust standard errors to account for potential clustering of patients between practices. | | | | | | |

eTable 24 presents risk differences per 100 patients treated and 95% confidence intervals for the relative association between prescription of varenicline compared to NRT and hypnotic/anxiolytic prescription, stratified by mental disorder, derived from instrumental variable models. There was evidence for an association between varenicline and reduced risk of hypnotic/anxiolytic prescription in patients with bipolar at 3 and 6-months follow-up, in patients with depression at 4-years follow-up, and in patients with neurotic disorder at 6-months follow-up. In the remaining groups/follow-ups there was no evidence of an association between varenicline and hypnotic/anxiolytic prescription, and there was no evidence for an association between varenicline and increased risk of hypnotic/anxiolytic prescription.
